# Negative reciprocal regulation between Sirt1 and Per2 modulates the circadian clock and aging

**DOI:** 10.1038/srep28633

**Published:** 2016-06-27

**Authors:** Rui-Hong Wang, Tingrui Zhao, Kairong Cui, Gangqing Hu, Qiang Chen, Weiping Chen, Xin-Wei Wang, Alejandro Soto-Gutierrez, Keji Zhao, Chu-Xia Deng

**Affiliations:** 1 Faculty of Health Sciences, University of Macau, Macau SAR, China; 2 Genetics of Development and Disease Branch, National Institute of Diabetes and Digestive and Kidney Diseases, National Institutes of Health, Bethesda, MD 20892, USA; 3 Systems Biology Center, National Heart, Lung, and Blood Institute, Bethesda, MD 20892, USA; 4 Genomic Core Laboratory, National Institute of Diabetes and Digestive and Kidney Diseases, Bethesda, MD 20892, USA; 5 Laboratory of Human Carcinogenesis, Center for Cancer Research, National Cancer Institute, National Institutes of Health, Bethesda, MD 20892, USA; 6 Department of Pathology, University of Pittsburgh, Pittsburgh, PA 15213, USA

## Abstract

Sirtuin 1 (SIRT1) is involved in both aging and circadian-clock regulation, yet the link between the two processes in relation to SIRT1 function is not clear. Using *Sirt1*-deficient mice, we found that *Sirt1* and *Period 2* (*Per2*) constitute a reciprocal negative regulation loop that plays important roles in modulating hepatic circadian rhythmicity and aging. *Sirt1*-deficient mice exhibited profound premature aging and enhanced acetylation of histone H4 on lysine16 (H4K16) in the promoter of *Per2*, the latter of which leads to its overexpression; in turn, Per2 suppresses *Sirt1* transcription through binding to the *Sirt1* promoter at the Clock/Bmal1 site. This negative reciprocal relationship between SIRT1 and PER2 was also observed in human hepatocytes. We further demonstrated that the absence of *Sirt1* or the ectopic overexpression of *Per2* in the liver resulted in a dysregulated pace of the circadian rhythm. The similar circadian rhythm was also observed in aged wild type mice. The interplay between Sirt1 and Per2 modulates aging gene expression and circadian-clock maintenance.

Human lifespan is on average about 25% heritable, but due to variations between individuals, it is not yet clear what genes contribute to this heritability^1^. Genes that experimentally cause shortened or prolonged lifespan largely function on central pathways that coordinate a variety of downstream mechanisms for maintenance and repair at multiple levels^2^,^3^. Thus, aging reflects a process of system failure. Furthermore, during this process, environmental factors play very important roles. Aging is thus a consequence of combined effects of environmental stress and genome specificity^4^,^5^.

The most widely studied environmental factor that affects aging is caloric restriction^6^. Sirtuins, a family of NAD^+^-dependent histone and protein deacetylases, have been shown to be regulated by caloric restriction. Because of this established relationship, it is important for aging studies to include examination of the role of sirtuins^7^. In humans, sirtuin 3 (SIRT3) has been shown to be important for longevity and to play a role in caloric restriction-induced lifespan extension^8^. Transgenic male mice that moderately overexpress *Sirt6* have been reported to display 14–15% longer lifespan^9^. Some members of the sirtuin family also play important roles in maintaining healthy lifespan through regulation of energy metabolism, genome integrity and tumor suppression^10^,^11^,^12^,^13^,^14^,^15^,^16^,^17^. Recent studies also uncover an important role of SIRT1 in regulating of circadian clock^18^,^19^,^20^.

The mammalian circadian clock, in both suprachiasmatic nucleus (SCN) and peripheral tissues, is established through positive transcriptional regulation of *CLOCK*/*BMAL1* and negative transcriptional regulation of *PERS*/*CRYS* in response to light and food stimulation^21^,^22^,^23^. CLOCK acetylates BMAL1 to enhance C*LOCK*/*BMAL1* transcriptional activity, while SIRT1 counteracts this by de-acetylating BMAL1 on the E-boxes^19^. It was shown that SIRT1 binds CLOCK-BMAL1 in a circadian manner and promotes the deacetylation and degradation of PER2 in the liver^18^,^19^. Using nestin-Cre mediated SCN-specific deletion of *Sirt1*, it was demonstrated that Sirt1, together with PGC1α, mediates central circadian control in SCN through positively regulating expression of circadian genes, especially *Bmal1*^20^. Sirt1 also negatively regulates transcription of several circadian-related genes by deacetylating histone H3 on lysine 9 (H3K9) of their promoters^24^.

While these previous studies demonstrate a critical role of SIRT1 in regulating circadian rhythms, the ways in which SIRT1 may connect the processes of aging and circadian rhythm remain elusive. In this study, we investigated this issue by using a cohort of *Sirt1*-deficient mice that undergo premature aging. We demonstrated that *Sirt1* and *Per2* display opposite circadian rhythms of gene expression and an inverse expression pattern throughout the aging process. Further analysis revealed that Sirt1 and Per2 constitute a reciprocal negative regulation loop that plays important roles in modulating circadian rhythmicity, metabolism, and aging.

## Results

### *Sirt1* Deficiency Leads to Premature Aging

**Figure 1:**
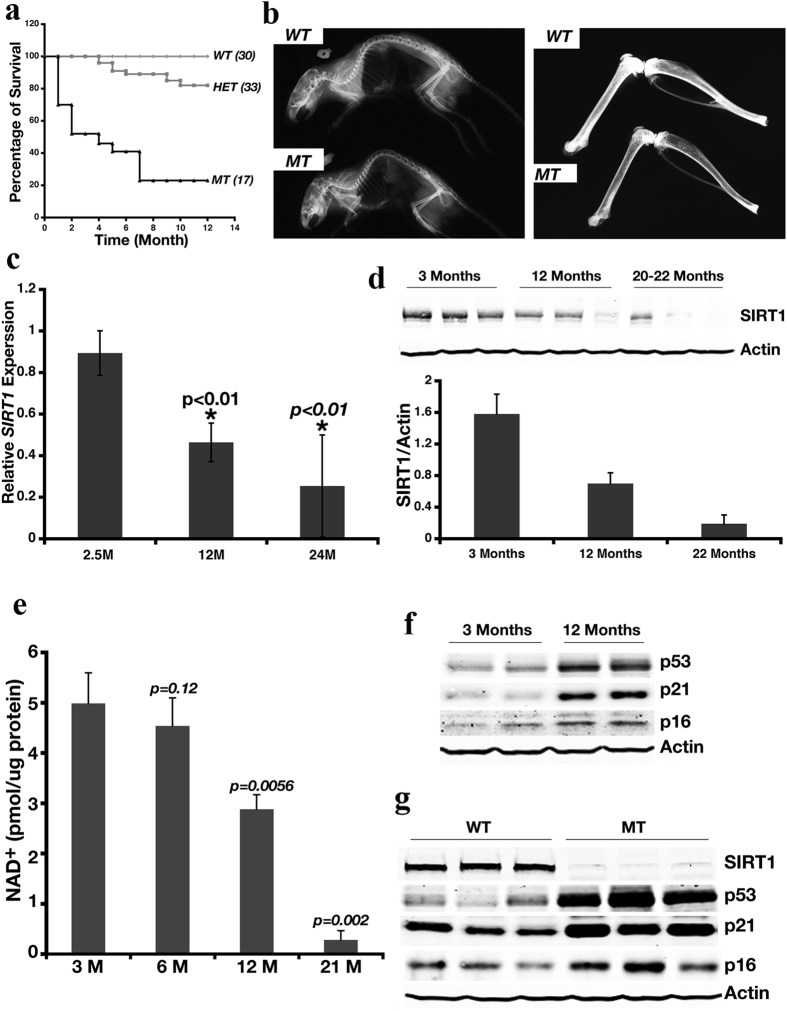
Deletion of *Sirt1* Leads to Premature Aging. (**a**) Kaplan-Meier curve for Survival of cohorts of wild-type (WT, n = 30), *Sirt1* heterozygous (HET, n = 33), and *Sirt1*-deficient mice (MT, n = 17). Among 17 MT mice, there were 7 females. Of 7 females, 1 died at 5 months, 2 died at 7 months and 4 dies at 9–12 months of age. (**b**) *Sirt1*-deficient mice display kyphosis (left panel) and decreased bone density (right panel). X-ray photos are representative of those obtained for 5 WT and MT mice analyzed. (**c**) qRT-PCR analysis of Sirt1 expression in livers of WT mice at different ages. Data represent the mean (± standard deviation [SD], n = 6). **p < 0.01 represents the comparison between 12 months vs 2.5 months and 19 months vs. 2.5 months respectively. The expression level of *Sirt1* in 2.5 months is set to 1. (**d**) Western blot analysis of Sirt1 protein in WT mice at different ages. Top panel, representative Western blot data; lower panel, quantitated Western blot data normalized to actin levels, mean (±SD, n = 6). (**e**) Mean NAD^+^ level (±SD, n = 5) in WT and MT mice at different ages. *p*-value obtained from the comparison between 3-month-old WT mice vs. all other groups of mice. (**f,g**) Western blot analysis of aging markers p53, p21, and p16 in WT mice at different ages (**f**) or in WT and MT mice at 3 months of age (**g**). Each group contained 5 mice; 2 mice from each time point are shown in (**f**) and 3 mice from each genotype are shown in (**g**).

We have previously shown that while the majority of *Sirt1*-deficient (*Sirt1*^−/−^) mice (in a genetic background of 129/FVB/Black Swiss at a ratio roughtly 1:2:1) that carry targeted deletion of exons 5–6 died during embryonic development, an average of 5% of them survived to adulthood^11^. To study the potential impact of *Sirt1* deficiency on mouse development, we followed 17 *Sirt1*^−/−^ (mutant, MT), 33 heterozygous (HET), and 30 wild-type (WT) mice over time. We observed that MT mice displayed a much shorter lifespan than HET and WT mice (Fig. 1a), and that 80% of these MT mice did not survive over a year. The MT mice appeared to suffer kyphosis and appeared much older than the WT mice of the same age (Supplementary Figure 1a). The MT mice weighed less than age-matched WT mice (Supplementary Figure 1b). In addition, peripheral blood chemistry analysis demonstrated sustained elevated numbers of granulocytes in MT mice compared with age-matched WT mice (Supplementary Figure 1c), indicating the presence of persistent inflammation. Although the granulocyte number increased over time in WT mice, the number of granulocytes in these mice was significantly lower than that found in age-matched MT mice (Supplementary Figure 1c). Further, MT mice displayed reduced bone density (Fig. 1b) and impaired wound-healing ability (Supplementary Figure 1d). Next, we examined these 2 genotypes of mice for β-galactosidase activity (an indicator for cellular senescence) and γH2AX foci (an indicator of DNA damage), which have previously been used as aging markers^25^,^26^. These markers were increased in the kidney, brain, liver, and intestine of MT mice compared with WT mice (Supplementary Figure 1e,f). Levels of hepatic Sirt1 transcripts and proteins progressively decreased from young to mid-age and aged WT mice (Fig. 1c,d). NAD^+^ levels also declined as WT mice aged (Fig. 1e), indicating diminished sirtuins activity. Reduced Sirt1 expression correlated with progressively increased protein levels of p53, p21, and p16 in the liver of WT mice (Fig. 1f), and levels of these markers were much higher in MT mice compared with WT mice (Fig. 1g). Increased expression of these genes suggests increased DNA damage and/or senescence. As aging process is accompanied with DNA damage, this data suggests that Sirt1 deficiency resulted in increased DNA damage and/or senescence, which is consistent with our data that Sirt1 mutant mice were short lived.

### Identification of Hepatic Sirt1-Dependent Aging-Related Genes

**Figure 2:**
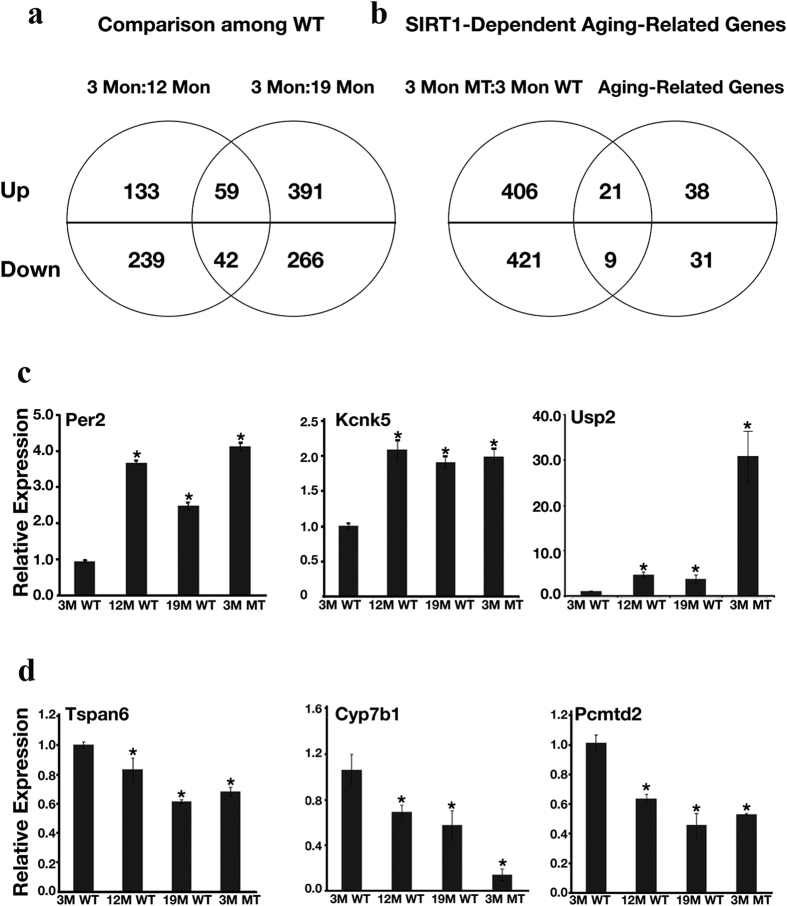
Identification of Hepatic *Sirt1*-Dependent Aging-Related Genes. (**a**) Venn diagram illustrates the number of aging-related genes generated from comparison between WT mice at different ages. Livers from 3 individual mice were used for microarray analysis for each set of samples. Gene expression from WT mice at 3, 12, and 19 months (Mon) of age were compared. Genes within the intersection of the 2 circles were defined as hepatic aging-related genes. (**b**) Venn diagram illustrates the number of Sirt1-dependent aging-related genes. Livers from 3 individual mice were used for microarray analysis for each set of samples. Gene expression from WT and MT mice at 3 months (Mon) of age was compared with each other, as well as with aging-related genes identified in (**a**). Genes within the intersection of the 2 circles were defined as Sirt1-dependent aging-related genes. (**c,d**) Representative validation results using qRT-PCR of up-regulated (**c**) and down-regulated (**d**) Sirt1-dependent aging-related genes. The level of gene expression for 3-month-old WT mice was set as 1. Data represent the mean ± SD (n = 3). *p < 0.05 when compared with WT 3-month-old samples.

We next wanted to investigate hepatic Sirt1-dependent aging-related genes. To do so, livers from WT mice at 3 months (young), 12 months (middle age), and 19 months (old) of age, as well as from MT mice at 3 months of age, were taken for microarray analysis. Three mice were utilized for each group, and the mice were sacrificed at 9–11 AM. Although 3-month old MT mice exhibited no obvious morphological signs of aging (data not shown), they still displayed high levels of some molecular aging markers (Fig. 1g). When we examined gene-expression profiles in WT mice of different ages by microarray analysis, we identified 192 upregulated and 281 downregulated genes in 12-month-old WT mice compared with 3-month-old WT mice, and 450 upregulated and 308 downregulated genes in 19-month-old WT mice compared with 3-month-old WT mice. We performed one more comparison to identify common genes in these 2 groups. This comparison identified 59 upregulated and 42 downregulated genes that showed consistent changes at both 12 months and 19 months in comparison with animals at 3 months of age (Fig. 2a). We named this group of genes as hepatic aging-related genes (Supplementary Table 1).

To define Sirt1-regulated genes in the liver, we compared the gene-expression profiles of 3-month-old WT and 3-month-old MT mice. This comparison revealed 427 up-regulated and 430 down-regulated genes in MT mice compared with WT mice of the same (young) age. We hypothesized that if Sirt1 is indeed involved in aging, then this group of differentially expressed genes should possess some common genes with our previously defined aging-related gene group. Thus, we next compared the Sirt1-regulated genes with the aging-related genes. This comparison revealed a statistically significant overlap of 21 up-regulated and 9 down-regulated genes (Fig. 2b). We named this group of genes as Sirt1-dependent aging-related genes (Supplementary Table 2). We confirmed expression changes of 14 out of 16 up-regulated genes and all 9 down-regulated genes by qRT-PCR analysis (Fig. 2c,d, and Supplementary Table 3).

### *Sirt1* Deletion Causes Global Enrichment of Ac-K16 of Histone H4

**Figure 3:**
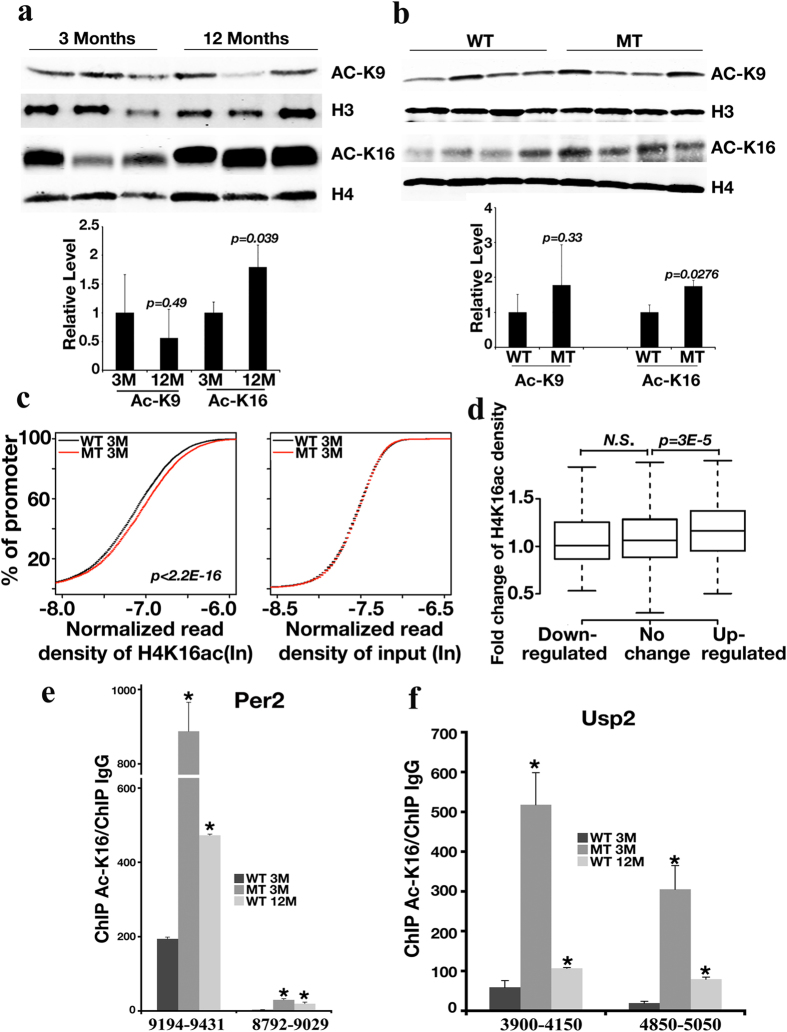
*Sirt1* Deletion Induces Global Increase of Ac-K16 of Histone H4. (**a,b**) Western blot analysis of histone H4K16 and H3K9 acetylation level in 3- and 12-month-old WT mice (n = 3) (**a**) and 3-month-old WT and MT mice (n = 4) (**b**). Upper panel is the representative image of western blot, the lower panel is the quantification of western blots. (**c**) Cumulative distribution of ChIP-Seq read density for H4K16ac at gene promoters for 3-month-old WT and MT mice. Livers from 2 pairs of mice were utilized for ChIP-Seq analysis. Y-axis shows the percentage of promoters that exhibit H4K16ac. A line shifted to the left means a systematically lower level of H4K16ac. *p*-value was calculated by the Kolmogorov-Smirnov test. The analysis was repeated by using input read densities for comparison purposes. (**d**) Boxplot of the fold-change of normalized H4K16ac densities between 3-month-old WT and MT mice at the promoter regions of genes up-regulated (150 genes), down-regulated (165 genes), and not changed (8937 genes) in the 3-month-old MT mice compared with the 3-month-old WT mice. The top and bottom of the box, and the band near the middle of the box represent 75th, 25th, and 50th percentiles, respectively. N.S., not significant. (**e,f**) Validation of H4K16ac enrichment in promoters of Sirt1-dependent aging-related up-regulated genes by qChIP. Data represent the mean (±SD, n = 3) *p < 0.05 when compared with WT 3-month-old samples. M, months; WT, wild-type; MT, mutant (*Sirt1*-deficient). qChIP data were first normalized with individual input, then normalized again with qChIP of IgG. Primer positions are shown in supplementary Figure 2.

We next explored if the gene expression changes observed during aging might be caused by alterations of Sirt1-mediated histone modification. When we examined the important hepatic histone acetylation sites, we found that histone H4K16 displayed increased acetylation in 12-month-old WT mice compared with 3-month-old WT mice, while no obvious change in acetylation of H3K9 was observed (Fig. 3a). Similarly, when the livers of 3-month-old WT and MT mice were compared, acetylation of H4K16 but not H3K9, was increased in MT mice compared with WT mice (Fig. 3b).

To detect changes at specific chromatin regions, we applied ChIP-Seq technology to perform a genome-wide analysis of H4K16ac in WT and MT mice. We observed a global increase in H4K16ac level at promoter regions in the 3-month-old MT mice (Fig. 3c, left panel and Supplementary Figure 2), however, we did not detect any difference from the input (Fig. 3c, right panel), suggesting that Sirt1 inhibits gene transcription by decreasing the histone acetylation levels in WT mice. Consistent with this idea, genes upregulated in the 3-month-old MT mice were associated with a greater increase in H4K16ac levels at their promoters than genes that were downregulated or unchanged (Fig. 3d), and this trend was not observed from the input controls (data not shown). We chose the top two genes (*Per2 and Usp2*) from our Sirt1-dependent aging related gene list to validate the increase of H4K16ac in 12-month-old WT mice and 3-month-old MT mice at their promoter regions by using ChIP-qPCR (Fig. 3e,f). Both *Per2 and Usp2* were found up-regulated in the *Sirt1*-deficient mice (Supplementary Table 3). As shown in Fig. 3e,f, the promoter regions for both genes contain higher acetylation levels on H4K16 when mice are old or *Sirt1* is deleted. Interestingly, genes down-regulated in the 3-month-old MT mice were not associated with a decrease in the H4K16ac level at their promoters (Fig. 3d), suggesting that these genes may be controlled by Sirt1 through other mechanisms.

### The *Per2* Promoter Is Subject to Epigenetic Modification during Aging

**Figure 4:**
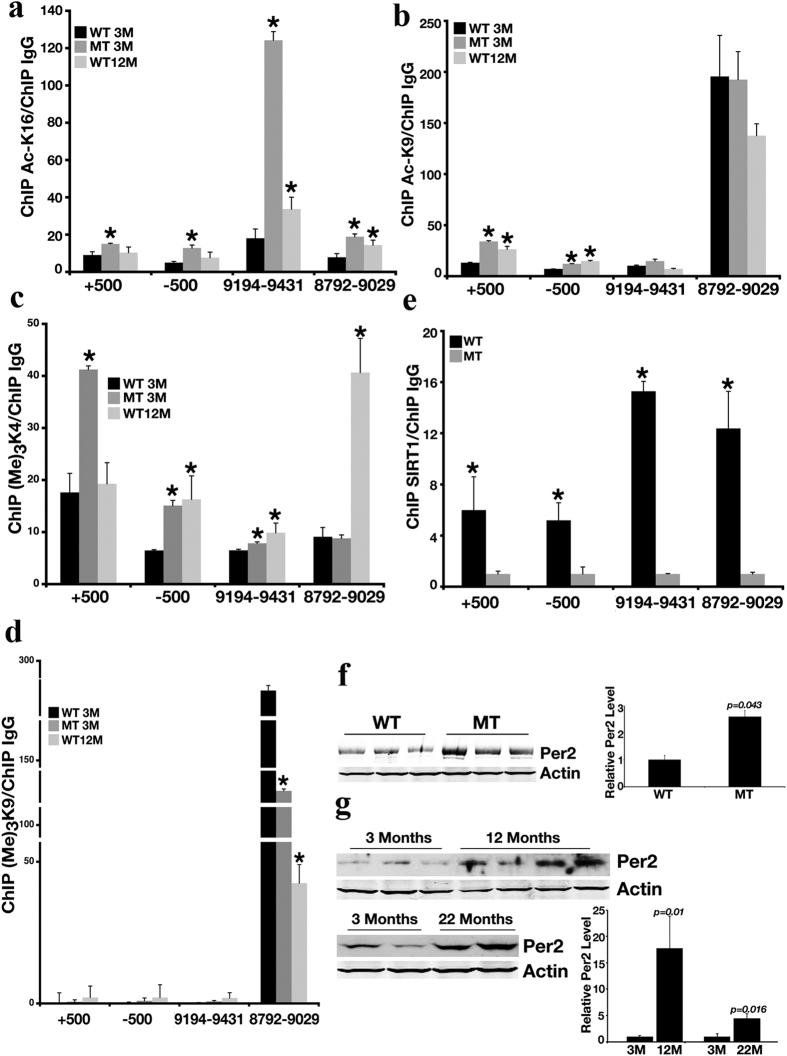
*Per2* Promoter Is Subject to Epigenetic Modification during Aging. (**a–d**) qChIP analysis of enrichment of H4K16ac (**a**), H3K9ac (**b**), H3K4(me)_3_ (**c**), and H3K9(me)_3_ (**d**) in the *Per2* promoter. All the data were normalized to the level of 3-month-old WT mice. *p < 0.05 when compared with 3-month-old WT mice. (**e**) qChIP analysis of Sirt1 occupancy on the *Per2* promoter. All the data were normalized to the level of 3-month-old MT mice. *p < 0.05 when compared with 3-month-old MT mice. For (**a–e**), data represent the mean (±SD, n = 3). Primer positions are shown in supplementary Figure 2. (**f,g**) Western blot analysis of Per2 protein levels in 3-month-old WT and MT mice (**f**) and during the aging process for WT mice (**g**). Each lane represents a liver sample from an individual mouse. The left panel is the representative image of western blot, the right panel is the quantification of western blots.

Of note, both USP2^27^ and PER2^18^,^19^,^20^ are involved in circadian clock regulation. We decided to first focus on the *Per2* gene, not only because it is one of the genes that is most upregulated during WT mouse aging, but also because previous studies have shown that the E-box of *Per2*′s promoter (+500 and −500) is regulated by histone H3 K9 acetylation^28^. Given the observed premature-aging phenotype of *Sirt1*-deficient mice, we were interested in determining whether the *Per2* promoter is subject to epigenetic modification during the aging process. We examined 2 candidate regions (−9194 to −9431 and −8792 to −9029) identified by our ChIP-Seq analysis (Supplementary Figure 2). Our data revealed significant enrichment of H4K16ac in these regions in both 12-month-old WT mice and 3-month-old MT mice compared with 3 months old WT mice (Fig. 4a), while no obvious changes were detected in histone H3K9 acetylation at these sites (Fig. 4b). These data suggest that H4K16ac increase is associated with aging, but H3K9ac is not.

Because acetylation and methylation are frequently intertwined, we also assessed trimethylation of H3K4 and H3K9 during aging of WT mice and in 3-month-old MT mice by regular ChIP. Trimethylation of histone H3K4, which is a marker for open-chromatin structure, was significantly enriched in the region of (−8792 to −9029) in 12-month-old WT mice, while its change was moderate or not observed in the other region (Fig. 4c). Trimethylation of histone H3K9, a marker for condensed chromatin, was reduced in region 8972 to −9029 in 12-month-old WT and 3-month-old MT mice (Fig. 4d). These data are consistent with the *Per2* expression pattern reported earlier.

Next, we studied Sirt1 occupancy on the *Per2* promoter in the liver of 3-month-old WT mice and data indicated that Sirt1 bound to the *Per2* promoter in both regions −9194 to −9431 and −8792 to −9029; such binding was not detected in the liver from 3-month-old MT mice (Fig. 4e). This is consistent with the idea that SIRT1 affects the histone H4K16 acetylation level in these regions. We further found that Per2 protein levels were markedly increased in the liver of 3-month-old MT mice (Fig. 4f) and in aged WT (12- or 22-month-old) mice (Fig. 4g) compared with 3-month-old WT mice. Altogether, these data indicate that the promoter of *Per2* is a target of Sirt1 during aging.

### PER2 Negatively Regulates SIRT1 Expression *in vitro* and *in vivo*

It has been reported that PER2 is a key molecule in maintaining the normal circadian clock^29^. The increased expression of Per2 in *Sirt1*-deficient mice that we observed prompted us to examine if Per2 could also affect Sirt1 expression. To investigate this, Sirt1 and Per2 expression patterns were examined in mouse liver samples collected according to Zeitgeber Time (ZT hour). Per2 and Sirt1 both oscillated in response to the circadian clock; however, the Sirt1 expression pattern was generally opposite to that of Per2 at both protein (Supplementary Figure 3a) and mRNA (Supplementary Figure 3b) level (i.e., when Sirt1 was high, Per2 was low, and vice versa). For instance, between ZT12 to ZT20 (Zeitgeber Time 12 hours to 20 hours), hepatic Per2 was high, but hepatic Sirt1 maintained its lowest level.

**Figure 5:**
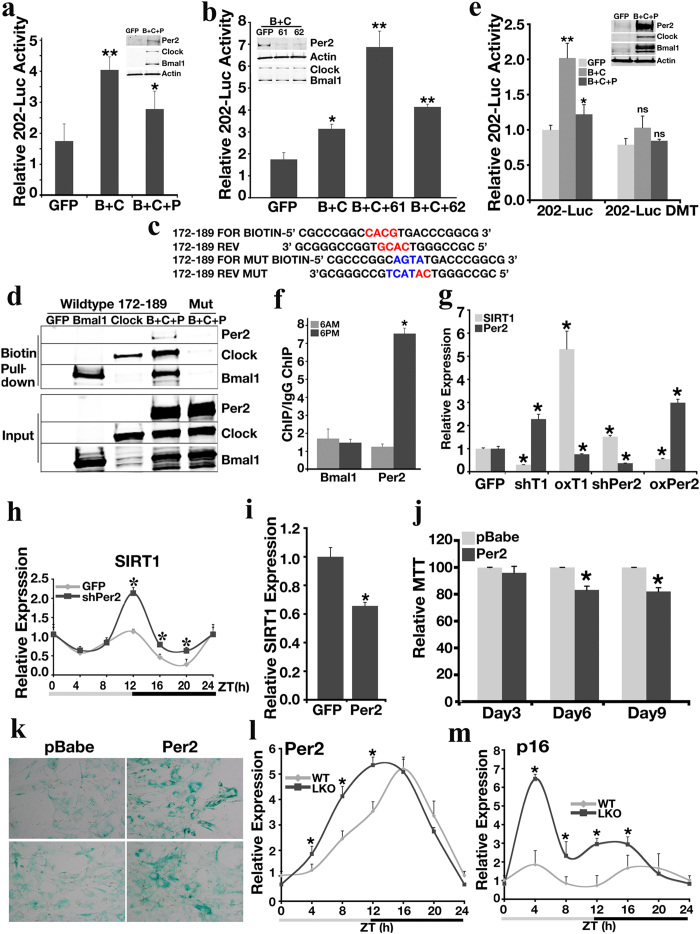
Sirt1 and Per2 Constitute a Reciprocal Negative Regulation Loop *in vitro* and *in vivo*. (**a**) *Sirt1* promoter luciferase reporter activity of 202-Luc construct following ectopic expression of *Bmal1* (**b**), *Clock* (**c**), and *Per2* (P) in MEFs. (**b**) Effect of *Per2* knockdown on *Sirt1* promoter 202-Luc construct luciferase reporter activity in response to *Bmal1* (**b**) and *Clock* (**c**) overexpression in MEFs. 61 and 62 are two independent shRNAs against *Per2*. (**c**) Biotin-labeled E-box sequences showing a possible Clock/Bmal1 binding site −(172–189). (**d**) Biotin pull-down assay examining Bmal1/Clock/Per2 binding to the *Sirt1* promoter at E-box region −(172–189). (**e**) *Sirt1* promoter 202-Luc luciferase reporter activity after mutation of the 2 E-boxes. DMT, mutant of two E-boxes core domains. The level of luciferase activity in GFP with WT 202-Luc cotransfected MEFs was set as 1. (**f**) ChIP analysis of Bmal1 and Per2 binding to the *Sirt1* promoter in the liver of 3-month-old WT mice collected at ZT0 and ZT12. *p < 0.05 when compared with ZT0 data. (**g**) qRT-PCR analysis of *SIRT1* and *PER2* expression in primary human hepatocytes (n = 2) subjected to shRNA knockdown of *SIRT1* (shT1) or *PER2* (shPer2) or overexpression of *SIRT1* (oxT1) or *PER2* (oxPer2). The level of expression for GFP vector-transfected cells was set as 1. (**h**) Hepatic *Sirt1* expression analyzed by qRT-PCR after 5 days of shPer2 virus infection. The level of expression of GFP-infected mice at ZT0 was set as 1. (**i**) *Sirt1* expression in MEF cells infected with retroviruses containing either Per2 or pBabe analyzed by qRT-PCR. (**j**) MTT assay in MEF cells infected with Per2 or pBabe retrovirus. (**k**) β-galactosidase activity assay in MEF cells infected with Per2 or pBabe retrovirus. (**l,m**) qRT-PCR analysis of *Per2* (**l**) and *p16* (**m**) expression in the livers of 3-month-old WT and Sirt1 liver specific knockout (LKO) mice obtained by circadian liver sample collection. Data represent the mean (±SD). Either 3 mice or MEFs were utilized for each experiment, and the experiment was repeated 3 times in a triplicated fashion. **p < 0.01, *p < 0.05.

To determine the key elements regulated by PER2 in the *Sirt1* promoter, we performed serial deletion of the *Sirt1* promoter and generated 5 constructs covering a 1.5 kb region upstream of the transcription initiation site. We found that fragment 202-Luc is the shortest construct that was both positively regulated by Clock/Bmal1 (Supplementary Figure 3c), and negatively regulated by Per2 (Supplementary Figure 3d). Co-transfection of *Clock*/*Bmal1* and *Per2* inhibited the ability of Clock/Bmal1 to activate 202-Luc (Fig. 5a). shRNA-mediated knockdown of *Per2* upregulated 202-Luc in response to *Bmal1* and *Clock* expression (Fig. 5b). Similar data were observed in the human hepatocellular carcinoma cell line, HepG2 (Supplementary Figure 3e).

*In silico* analysis detected 2 possible Clock/Bmal1 binding E-box sites [−(100–116) and −(172–189)] upstream of ATG in the *Sirt1* promoter (Supplementary Figure 3f and Fig. 5c). Using a biotin pull-down assay, we verified that Clock/Bmal1/Per2 bound to both sites, but displayed stronger binding ability to −(172–189). Mutation of the core domains of these 2 E-box sites abolished the binding of the Clock/Bmal1/Per2 complex (Fig. 5d and Supplementary Figure 3g). We also performed site mutagenesis on the *Sirt1* promoter 202-Luc construct, and then analyzed luciferase activity in response to *Clock*/*Bmal1*/*Per2* overexpression. Mutation of the E-box cores abolished the stimulatory activity of Clock/Bmal1 on 202-Luc, as well as the inhibitory effect of Per2 on this promoter region (Fig. 5e), confirming that the Clock/Bmal1/Per2 complex binds to the −(100–116) and −(172–189) regions within the *Sirt1* promoter.

ChIP analysis of Per2 and Bmal1 was performed in WT mouse liver samples to detect if the binding of these 2 molecules to the *Sirt1* promoter depends on a circadian rhythm. Bmal1 binds to the *Sirt1* promoter in the light/dark cycle, but Per2 binds to the *Sirt1* promoter more strongly at night (Fig. 5f), which is when Sirt1 expression is at its lowest level (Supplementary Figure 3a,b). In mouse embryonic fibroblasts (MEFs), the circadian complex of Clock/Bmal1/Per2 bound to the *Sirt1* promoter in the same fashion following serum shock (Supplementary Figure 4a). Sixteen hours post-serum shock, the binding of Per2 to the *Sirt1* promoter was significantly increased at the region of Clock/Bmal1 binding site. This increased level of binding correlated with a low wave of *Sirt1* expression (Supplementary Figure 4b). Deletion of *Sirt1* enhanced expression of many circadian-related genes, such as Per1, Per2 and Cry2 (Supplementary Figure 4c).

Next, we investigated if the negative reciprocal regulation between SIRT1 and PER2 exists in human primary hepatocytes. In two different preparations of freshly isolated human hepatocytes, knockdown of *SIRT1* upregulated endogenous *PER2* expression; overexpressing *SIRT1* decreased *PER2* expression. Meanwhile, knockdown of *PER2* elevated endogenous *SIRT1* expression, and overexpression of PER2 diminished *SIRT1* expression (Fig. 5g). We also performed the same analysis in the hTERT-immortalized human primary hepatocyte cell line HHT-4^30^, and a similar relationship between the two genes was detected (Supplementary Figure 4d).

When shPer2 lentivirus was injected into wild type mice through tail vein, *SIRT1* expression is elevated at multiple circadian points during dark cycle (Fig. 5h). When SIRT1 was overexpressed in WT MEF cells, acetylation of H4K16 on *Per2* promoter is significantly reduced (Supplementary Figure 4e); knocking down SIRT1 in WT MEF cells enhance acetylation of H4K16 on *Per2* promoter (Supplementary Figure 4f). These data demonstrated that a negative reciprocal relationship between SIRT1 and PER2 exists in both human and mouse hepatic systems.

We showed earlier that multiple tissues in *Sirt1*-deficient mice displayed senescence (Supplementary Figure 1e). Based on these initial findings, we wanted to explore if dysregulation of Per2 could influence senescence. To do so, we used retroviruses containing Per2, or empty pBabe vector to infect primary WT MEF cells. Overexpression of *Per2* decreased endogenous *Sirt1* levels in MEF cells (Fig. 5i). Six days post-infection, cell growth was significantly decreased in *Per2*-infected cells (Fig. 5j). β-galactosidase activity shown by x-gal staining demonstrated that much more cells overexpressing *Per2* had entered senescence than the control cells (Fig. 5k).

To further investigate the relationship among *Sirt1*, *Per2* and senescence in a circadian fashion, we utilized liver-specific *Sirt1* knockout mice (*SIRT1LKO*). Liver samples were collected based on the Zeitgeber Time (ZT) and gene expression was analyzed by qRT-PCR. The deletion of *Sirt1* in the liver disturbed *Per2* circadian expression pattern when compared with WT controls (Fig. 5l). Absence of *Sirt1* also dramatically affected the expression rhythm over the light/dark cycle of some other genes, such as *p16* (Fig. 5m), *Cry1* (Supplementary Figure 4g), *p19*, and *Pepck* (Supplementary Figure 4h). These genes play important roles in aging, circadian rhythmicity and glucose metabolism. Taken together, the above data indicate that SIRT1 and PER2 constitute a reciprocal negative regulation loop and regulate aging process both *in vitro* and *in vivo*.

### Overexpression of *Per2* Represses *Sirt1* Expression, Impairs Circadian Rhythm, and Increases Expression of Aging Markers

**Figure 6:**
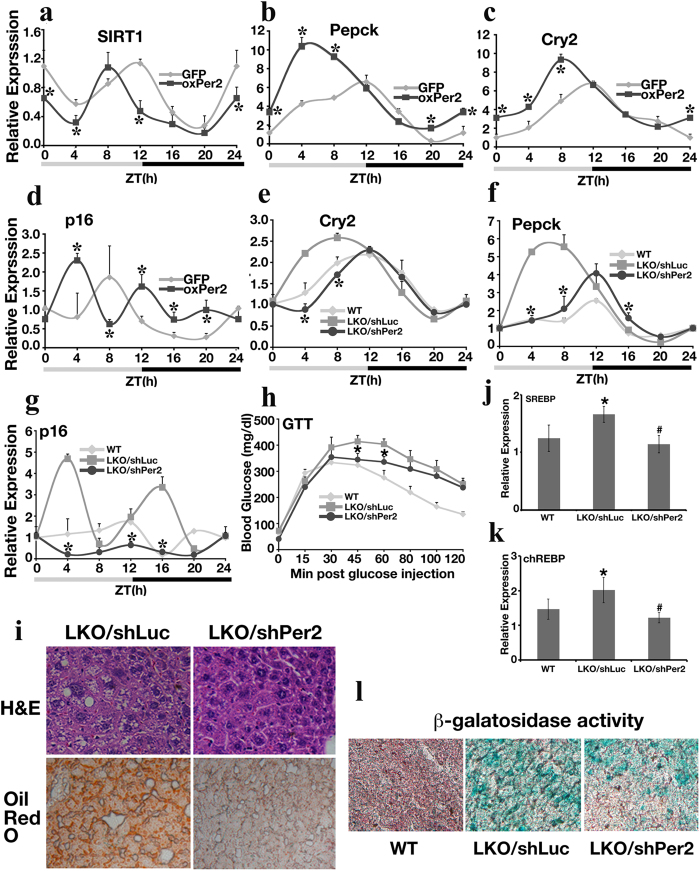
Overexpression of *Per2* in Mouse Liver Disrupts the Circadian Clock and Causes Increased Expression of Aging Markers. (**a–d**) Hepatic gene expression analyzed by qRT-PCR after 5 days of Per2 virus infection. The level of expression of GFP-infected mice at ZT0 was set as 1. *p < 0.05 when compared with GFP infected group. (**e–g**) qRT-PCR analysis of hepatic gene expression 5 days post-shPer2 virus infection. WT: wild-type; LKO/shLuc: SIRT1LKO mice infected with shLuciferase lentivirus; LKO/shPer2: SIRT1LKO mice infected with shPer2 lentivirus. The level of gene expression of the WT mice at ZT0 was set as 1. *p < 0.05 when LKO/shPer2 group was compared with LKO/shLuc group. Each dot represents average data from 3 individual mice. In total, each curve contains liver samples from 21 mice. (**h**) Glucose tolerance test (GTT) in 3-month-old male WT, LKO/shLuc, and LKO/shPer2 C57BL/N6 mice. *p < 0.05 when LKO/shPer2 group was compared with LKO/shLuc mice at the same time point. Ten mice from each genotype were used for this analysis. (**i**) H&E and Oil Red O staining on liver sections from shLuciferase and shPer2 lentiviurs infected mice. 3 mice at age of 9-month from each genotype were analyzed. (**j,k**) The expression level of lipid synthesis genes SREBP (**j**) and chREBP (**k**) was analyzed by qRT-PCR. Liver samples of mice from panel I were utilized, and *p < 0.01 represents the comparison between LKO/shLuciferase and WT; ^#^p < 0.01 represents the comparison between LKO/shLuciferase and LKO/shPer2. (**l**) β-galactosidase activity assessed by x-gal staining in 3 month-old mice livers. 3 mice from each genotype were analyzed. All mice used in this figure were males in a C57BL/6N background. Mice for panel A–H and L are 3 months old males.

To provide evidence that *Per2* may regulate *Sirt1* levels *in vivo*, we injected virus for Per2 into the tail vein of 3-month-old mice and collected liver samples 5 days post-injection based on the circadian cycle. Overexpression of *Per2* (Supplementary Figure 5a) substantially reduced *Sirt1* expression at multiple time points during the ZT cycle (Fig. 6a) and influenced the expression of *Sirt1* downstream genes *Pepck* (Fig. 6b) and *G6pase* (Supplementary Figure 5b), which are known to be regulated by *Sirt1* and other circadian transcription factors, such as BMAL1/CLOCK. The gluconeogenesis genes *Pepck* and *G6pase* both displayed elevated expression levels in the Per2 overexpressed mice when compared with GFP retrovirus-infected mice at multiple time points, especially at ZT0, 4, 8 h. Overexpressing *Per2* also altered the *Cry2* circadian rhythm pattern (Fig. 6c) with advanced phases, and elevated the expression of aging-related genes *p16* (Fig. 6d) and *p19* (Supplementary Figure 5c) at several time points. This evidence suggests that overexpression of *Per2* disrupts circadian oscillation, reduces *Sirt1* levels, alters *Sirt1* downstream gene expression, and increases expression of senescence markers, all of which may contribute to the shortened lifespan observed in *Sirt1*-deficient mice.

### Knocking Down Per2 Improves Aging Phenotype in *SIRT1LKO* Mice

Next, we investigated whether reducing *Per2* in *SIRT1LKO* mice would be able to correct the impaired glucose metabolism and the expression of aging genes. Knockdown of *Per2* with shPer2 lentivirus reduced hepatic Per2 mRNA and protein levels efficiently in *SIRT1LKO* mice (Supplementary Figure 5d). Under these conditions, the rhythm of circadian gene expression for *Cry2* shifted towards to the pattern that was observed in WT mice (Fig. 6e). At the same time, gluconeogenesis genes Pepck (Fig. 6f) and G6pase (Supplementary Figure 5e) in shPer2 lentivirus-infected LKO mice also showed a rhythmic pattern that is closer to that of wild-type mice. The level of the aging markers *p16* (Fig. 6g) and *p19* (Supplementary Figure 5f) was dramatically reduced at multiple time points after *Per2* knockdown. In addition, SIRT1LKO/shPer2 mice displayed improved glucose tolerance ability during a glucose tolerance test (Fig. 6h); decreased hepatic oil droplet formation (Fig. 6i), and reduced expression of lipid synthesis genes SREBP (Fig. 6j) and chREBP (Fig. 6k). Notably, knockdown of *Per2* also markedly reduced cell senescence revealed by decreased β-galatosidase activity (Fig. 6l). These data confirm that the negative reciprocal regulation loop between Sirt1 and Per2 observed *in vitro* also occurs *in vivo* and alteration of Per2 in *SIRT1LKO* mice contributes to abnormal circadian oscillation and premature aging phenotypes.

## Discussion

In this study, by comparing *Sirt1*-deficient and WT mice, we have provided a link between the processes of aging and the circadian clock. We have shown that both natural aging and *Sirt1* deficiency-induced aging involve alterations in circadian rhythms and amplitude of the expression of *Per1*, *Per2*, *Cry1* and *Cry2* genes, and, furthermore, that the alterations in *p16* and *p19* expression present in the normal aging process are accelerated by *Sirt1* deficiency. We further demonstrated that Sirt1 and Per2 negatively regulate each other and exhibit an inverse expression pattern during the natural aging process.

Previous studies have suggested that *Sirt1* plays an important role in aging, yet some controversies remain^31^,^32^,^33^,^34^. For example, overexpression of the *Sir2* ortholog in budding yeast, *C. elegans*, and *Drosophila* has been reported to increase lifespan^35^. However, a recent study indicated that the reported effect of lifespan extension in yeast and *Drosophila* is attributed, at least in part, to background mutations^36^. In mice, whole-body *Sirt1* overexpression^37^ or activation of *Sirt1* by SRT1720^38^ did not increase lifespan, although it did improve metabolic conditions. More recently, it has been shown that *Sirt1* overexpression specifically in the brain improved healthy lifespan and delayed aging in mice^39^. These effects were mediated by enhanced neural activity specifically in the dorsomedial and lateral hypothalamic nuclei, through increased expression of orexin type 2 receptor. Thus, while all these studies support a critical role of *Sirt1* in contributing to a healthy lifespan, its effect in extending lifespan is complex.

Three *Sirt1* mutant mouse lines reported so far^40^,^41^ displayed some phenotypic variations either because of their different genetic backgrounds or nature of mutations, or a combination of both, as discussed previously^11^. The *Sirt1*^−/−^ mice used in this study exhibited most severe phenotype with a majority of them died at middle gestation and the small fraction of survivors suffered from premature aging, characterized by markedly increased expression of *p53*, *p21*, and *p16*, as well as widespread cellular senescence and DNA damage. The premature aging associated with *Sirt1* deficiency can be attributed to a combination of several mechanisms. First, Sirt1 is known to destabilize p53 by deacetylating it at Lys382^42^,^43^; therefore, the absence of Sirt1 may contribute to the increased levels of p53 and its downstream gene *p21*. Because increased expression of p53 or p53 activation in mice eventually causes premature aging^26^,^44^, it is conceivable that the premature aging observed here is, at least in part, mediated by *p53* overexpression/activation. Secondly, *Sirt1* plays important roles in organismal metabolism and genome integrity^32^,^45^,^46^, which are essential for the maintenance of the healthy lifespan. *Sirt1* deficiency may cause malfunctions of many organs/tissues and accumulation of unrepaired DNA, and collapse of DNA replication forks^11^, leading to premature aging. Thirdly, we found that *Sirt1* deficiency caused abnormalities in the circadian clock, which may produce a profound impact on aging.

The circadian clock has been well studied in the liver. In response to light and food stimulation, CLOCK acetylates BMAL1, which in turn collaborates with some other factors to activate transcriptions of many downstream genes^21^,^22^,^23^,^47^,^48^. SIRT1 counteracts this action by binding and de-acetylating BMAL1 in a circadian manner and promotes the deacetylation and degradation of PER2^18^,^19^,^21^,^22^,^23^,^47^,^48^. Our study advances this understanding by demonstrating that Per2 also negatively regulates *Sirt1* transcription, and Sirt1 and Per2 constitute a negative regulation loop that affects both the amplitude and pace of circadian rhythms. Per2 is a key molecule in maintaining the normal circadian clock^21^,^22^,^23^,^49^. It has previously been shown that Sirt1 deacetylates H3K9 in the E-box region of *Per2* promoter, resulting in reduced transcription^28^. We showed a more robust action of Sirt1 to deacetylate H4K16 than H3K9 in the *Per2* promoter region to decrease its transcription. We demonstrated that the *Per2* promoter is subject to epigenetic modification during aging. With an increase in age, the *Per2* promoter displayed a more open state and increased transcription, which is correlated with reduced expression of *Sirt1*. On the other hand, Per2 also suppressed *Sirt1* transcription through binding to the promoter at the Clock/Bmal1 binding site. Thus, the two genes exhibited an inverse expression pattern during aging. Furthermore, we found that *Sirt1* deletion or *Per2* overexpression led to increased expression of signature aging genes, such as *p16*, *p19*, *p21*, and *p53*, and an advanced pace of circadian rhythm, both of which mimic the expression patterns observed in natural aged mice. Circadian clock plays important roles in energy metabolism and organismal homeostasis^50^, which may account for the involvement of Sirt1-Per2 axis in regulation of aging. Thus, our study uncovers an important interplay between Sirt1 and Per2 that modulates the mechanisms of aging and the circadian clock.

**Figure 7:**
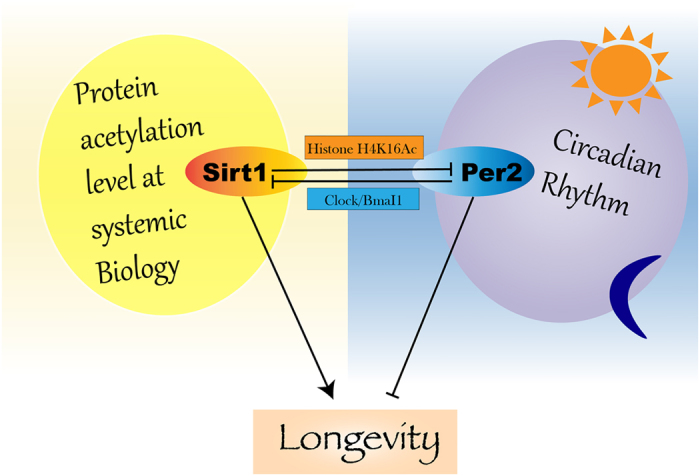
Diagram to show the relationship between Sirt1 and Per2 in regulating aging process. Sirt1 is involved in many biological processes through affecting protein acetylation dynamics, thus, promotes longevity. By deacetylating histone H4 K16 on Per2 promoter, Sirt1 downregulates Per2 expression. In return, Per2 inhibits Sirt1 expression by shutting down Clock/Bmal1 activation on Sirt1 promoter. Per2, as an important negative regulator in Circadian, its upregulation causes premature aging. Hence, losing the balance of reciprocal inhibitory relationship between Sirt1 and Per2 is a major step that leads to dysregulation of Sirt1 dependent aging progress.

From our study and other reports, Per2 is an important molecule that is regulated at multiple layers by Sirt1 in aging process (Fig. 7). During aging, as Sirt1 level is reduced, Per2 promoter gradually adapts an open state due to increased level of acetylation of histone H4 K16, which leads to the increase of Per2 transcription. At the same time, reduction of Sirt1 activity also allows Per2 protein stays in acetylated state, which stabilizes per2 protein and keeps it active as a transcription repressor. Interestingly, mice carrying overexpression of intact Per2 displayed shortened circadian rhythm, which is consistent with what we observe here in this report.

While aging and the circadian clock affect each other, the age-associated changes in circadian rhythms are rather complex. It has been reported that circadian transcriptional rhythms can be differentially regulated in different tissues^51^,^52^. It can also be affected by different strain background^53^, and genders^54^. Therefore, the comparisons of gene expression were made only in same genetic background and same gender (see Supplementary online methods for more details). Of note, a recent study using an adult-inducible *Sirt1* knockout mouse (SIRT1-iKO) indicated that loss of *Sirt1* significantly decreased NAD^+^ levels, which might contribute to animal aging through triggering increased expression of HIF-1α and its target genes in the muscle^55^. Hence, we examined levels of HIF-1α and its downstream genes in the liver of WT and *Sirt1*-deficient (MT) mice. Our analysis did not reveal obvious differences in expression levels of HIF-1α in WT mice during circadian-clock progression and the aging process, or between WT and MT mice at 3 months of age (Supplementary Figure 6a). HIF-1α downstream gene expression also showed no detectable change during the aging process in WT mice or between WT and MT mice at 3 months of age (Supplementary Figure 6b). Furthermore, although we found significantly decreased NAD^+^ levels in older (≥12-months-old) WT mice, we detected no statistically significant change of NAD^+^ levels between WT and MT mice at 3 months of age (Fig. 1e), when profound molecular differences between the two genotypes had already become obvious. Thus, NAD^+^ /HIF-1α may not play an active role in the hepatic aging process although their important role in the muscle, supporting the notion that circadian clock is subject to differential regulation in different tissues when an organism is undergoing the aging process^51^,^52^.

## Methods

### Mouse and human samples

Mouse experiments were performed in accordance with the guidelines of Institutional Animal Care and Use Committee (IACUC). All animal experimental protocols were approved by either the Animal Care and Use Committee of the National Institute of Diabetes and Digestive and Kidney Diseases or the Animal Care and Use Committee of University of Macau. The usage of human samples were carried out in accordance with the guidelines of NIH Human Research Protections Program (HRPP). The de-identified normal human liver cells were obtained through the Liver Tissue Cell Distribution System (Pittsburgh, PA) after obtaining a written informed consent by a protocol approved by the Human Research Review Committee of the University of Pittsburgh, which was funded by NIH Contract # HSN276201200017C.

Male and female *Sirt1* mutant mice (*Sirt1*^+/−^)^11^ were intercrossed to generate *Sirt1*^−/−^ (MT), *Sirt1*^+*/−*^ (HET), and *Sirt1*^+/+^ (WT) mice. Because difference in genetic background and gender might affect gene expression, we only compared gene expression within the same genetic background and the same gender (i.e. comparisons were made either among males or females in same genetic background). Specifically, the mutant mice used in Figs 1c–g and 2, 3, 4 were males in a mixed genetic background of 129/FVB/Black Swiss at a ratio roughly 1:2:1. In Figs 5h,l,m and 6, when large quantity of Sirt1 mutant mice (24 mutant mice were needed for each curve) was required for circadian studies after gene knockdown or over-expression, which could not be performed using Sirt1-deficient mice due to their poor viability, all comparisons were made using Sirt1-liver specific knockout mice (*Sirt1LKO*) in C57BL/6N background. To match up with this, all control mice were littermates in the same background.

Mice were maintained in a 12-h light/12-h dark cycle environment. The mouse cohorts were followed for 2 years. Because the survival rate for MT mice is very low, no any inclusion and exclusion criteria were used when MT mice were selected for use in experiments. The WT and HET mice appeared normal and were randomly picked for experiments. All animal experiments were approved by the Animal Care and Use Committee of the National Institute of Diabetes and Digestive and Kidney Diseases.

### X-ray analysis

Mice were anesthetized with Avertin. Whole mice or the dissected femur/tibias were placed into a Faxitron X-ray apparatus (Specimen Radiography System) and BioMax XAR X-ray film (Kodak) was used to capture images.

### β-galactosidase activity assay

Tissues were embedded in OCT compound, and 10 μm cryosections were prepared and fixed with 1% formalin. Mouse embryonic fibroblasts (MEFs) were fixed with 0.5% glutaraldehyde for 5 min at room temperature. Slides were then incubated at 37 °C overnight with a solution containing K_4_ [Fe(CN)_6_] 3H_2_O and K_3_[Fe(CN). Afterwards, the slides were counterstained with FastRed.

### ChIP-Seq and Analysis

Mouse livers were harvested at 9 AM–11 AM. The fresh liver pieces were snap frozen with liquid nitrogen on site. Later, about 1 mg of snap-frozen liver piece were pulverized in the presence of liquid nitrogen, and the dissociated tissue was first cross linked with 1% formaldehyde. Chromatin was prepared from these formaldehyde-crosslinked tissues and fragmented to an average size of 200 bp by sonication. ChIP-Seq experiments were performed as described previously^56^ using antibodies against H4K16AC (Santa Cruz). Briefly, 2 μg of the antibodies bind to 20 μl of Dynabeads protein A beads at room temperature for 1 hr with rotation. Add chromatin mixture from 1 mg liver tissue to the beads + antibody, incubate at 4 °C for overnight with rotation. Then wash, reverse cross-link, precipitated ChIP DNA.

The preparation of sequencing library was conducted with the following steps modified from^56^: (1) The ChIP DNA ends were repaired by using the Epicentre DNA END-Repair kit (Cat. No. ER0720, Epicentre Biotechnologies). (2) “A” was added to 3′ of DNA end by using Klenow Fragment (3′− > 5′ exo-) from NEB. (3) Illumina adaptor was ligated to the DNA end by T4 DNA Ligase. (4) The ChIP DNA was amplified by using illumine index primers. (5) ChIP DNA was isolated from agarose gel. (6) The purified DNA was used directly for illumine sequence.

### Circadian liver sample collection

The mice were housed in a 12-h light/12-h dark cycle environment. During circadian-clock experiments, the livers were collected every 4 h according to the light cycle and snap-frozen with liquid nitrogen until further processing. Samples were designated according to the number of hours elapsed from the beginning of the light cycle (e.g., ZT4 represents the sample that was collected 4 h after the start of the light cycle). For Figs 5h,l,m, 6 and 7, each time point contains 3 individual mice, and each line contains 24 mice.

### Lentivirus/Retrovirus injection

C57BL/6N male mice at 3 months of age were tail vein injected with virus at 1 × 10^8^ virus/200 μl PBS. Viruses used were: GFP retrovirus; shLuciferase (shLuc) lentivirus; shPer2 lentivirus; pBabe retrovirus, or Per2 retrovirus. Injections were performed twice, one week apart. The shLuciferase and shPer2 lentivirus vectors were purchased from Open Biosystems. Per2 vector was obtained from Addgene. Each virus was produced in HEK293T cells according to the manufacturer’s instructions. Virus purification was performed by ultracentrifugation.

### Quantitative RT-PCR (qRT-PCR)

Total RNA was isolated with STAT-60^TM^ (Tel-Test, Inc.) from livers or MEF cells. cDNA was synthesized with QuantiTect Reverse Transcription Kit (QIAGEN). qRT-PCR was performed using a SYBR green PCR Master Mix (Applied Biosystems, Roche) and 7500 Real Time PCR (Applied Biosystems). Primer sequences are listed at the end of the Methods section. Mouse Sirt1 and 18S primers have been published before.

### Luciferase activity assay

pGL3B vector containing different promoter sequences were transfected into primary MEF cells. All transfections were performed with Lipofectamine^TM^ 2000 (Invitrogen). After a 24-h incubation, luciferase activity was assessed with the Dual-Luciferase Reporter Assay Kit (Promega). The shRNAs against Per2 (61 and 62) were purchased from Open BioSystems. Myc-Per2, Myc-Clock, and Myc-Bmal1 plasmids were obtained from Addgene.

### qChIP

Livers or MEF cells were cross-linked with 1% formalin for 15 min and ChIP was performed with Sirt1, Per2, Kcnk5, Bmal1, H4K16ac, H3K9ac, H3K4(Me)_3_, or H3K9(Me)_3_ antibodies as described previously^57^. Primer sequences are listed at the end of the Methods section.

### Biotin-streptavidin pull-down assay

Four oligonucleotides containing biotin on the 5′-nucleotide of the sense strand were used in the pull-down assays. One microgram of each double-stranded oligonucleotide was incubated with 300 μg of nuclear protein extracts for 20 min at room temperature. Streptavidin-agarose beads (30 μl, Sigma-Aldrich) that had been pre-absorbed by poly(dI-dC) (Sigma-Aldrich) were added and incubated with the extract for 4 h at 4 °C. The protein-DNA-streptavidin-agarose complexes were pelleted by centrifugation and analyzed by Western blotting with Myc-tag antibody.

### Mutagenesis

To mutate the core E-box domains of 202-Luc, 202-pGL3B plasmid was used as a parental template. The PCR method (PCR SuperMix High Fidelity, Invitrogen) was applied and mutation was confirmed with sequencing. Mutagenesis was performed by BioInnovatise Inc. (Rockville, MD).

### Microarray

Mouse livers were harvested at 9 AM–11 AM. Fresh liver samples were immersed in RNALater (Ambion) overnight. Afterwards, the samples were extracted with STAT-60^TM^ (Tel-Test, Inc.) to retrieve total RNA. The RNA was purified further with RNeasy^R^ Mini Kit (QIAGEN). An RNA Analyzer (Agilent) was used to check RNA quality. RNAs with high quality were reverse transcribed and the cDNAs were labeled and hybridized to the Affymetrix GeneChip mouse genome 2.0 array. Gene expression data generated by the Affymetrix MOE430 chip (two samples per condition) were imported to GeneSpring for analysis. Absence and presence call for each probe was made by MAS5. To identify differentially expressed genes between any 2 conditions (fold-change >1.5 and *p*-value < 0.05; *t*-test), probes labeled by absence on both duplicates in either condition were discarded.

### NAD^+^ /NADH quantification

NAD^+^ levels were assessed with the NAD/NADH Assay Kit (Abcam, Cat. No. ab65348) according to the manufacturer’s instructions.

### Cell culture and treatment

Primary MEF cells were derived from E14.5 embryos using a standard procedure. Cells were maintained in DMEM with 15% fetal bovine serum. For serum-shock assays, 5 × 10^5^ MEF cells were seeded into a 10-cm dish and cultured in DMEM with 15% fetal bovine serum for 7 days until confluence, then 50% horse or calf serum was used to shock the cells for 2 h. The cells were then release to normal culture condition. After 12 hours, the samples were collected every 4 h. HepG2 cells were obtained from the American Type Culture Collection and grown in DMEMde. HHT-4 cells were provided by Dr. Xin-Wei Wang and grown in described condition. All cell lines were tested and free for mycoplasma contamination.

### Statistical analysis

Student’s *t*-test or two-way Anova (Prism) was used for analyzing the significance among different set of samples. Briefly, data (triplicated) obtained from at least 3 individual biological samples for each genotype at multiple time points were input into online student t-test calculation table (http://www.physics.csbsju.edu/stats/t-test_bulk_form.html), afterwards, p value was calculated. When p value is <0.05, the comparison is considered significant. For ChIP-Seq analysis, *p*-value was calculated by the Kolmogorov-Smirnov test. The analysis was repeated by using input read densities for comparison purposes. For Microarray, gene expression data generated by the Affymetrix MOE430 chip (two samples per condition) were imported to GeneSpring for analysis. Absence and presence call for each probe was made by MAS5. Differentially expressed genes were identified between any 2 conditions (fold-change >1.5 and *p*-value < 0.05; *t*-test), probes labeled by absence on both duplicates in either condition were discarded.

The ChIP-Seq and microarray data sets for this study have been submitted to NLM/NCBI. The data are available at http://www.ncbi.nlm.nih.gov/geo/query/acc.cgi? token=kjohyouybhefnip&acc=GSE54451.

## Additional Information

**How to cite this article**: Wang, R.-H. *et al*. Negative reciprocal regulation between Sirt1 and Per2 modulates the circadian clock and aging. *Sci. Rep.* **6**, 28633; doi: 10.1038/srep28633 (2016).

## Supplementary Material

Supplementary Information
