# Supplementary material for: Negative reciprocal regulation between Sirt1 and Per2 modulates the circadian clock and aging
Source: Sci Rep. 2016 Jun 27;6:28633. doi: 10.1038/srep28633 (PMC4922021; doi:10.1038/srep28633)
Supplement: Supplementary Information [file srep28633-s1.pdf]

Negative reciprocal regulation between Sirt1 and Per2 modulates the circadian clock and aging

Rui-Hong Wang[1,2], Tingrui Zhao[2], Kairong Cui[3], Gangqing Hu[3], Qiang Chen[1], Weiping Chen[4], Xin-Wei Wang[5], Alejandro Soto-Gutierrez[6], Keji Zhao[3], and Chu-Xia Deng[1, 2]*

[1]Faculty of Health Sciences, University of Macau, Macau SAR, China, [2]Genetics of Development and Disease Branch, National Institute of Diabetes and Digestive and Kidney Diseases, National Institutes of Health, Bethesda, MD 20892, USA, [3]Systems Biology Center, National Heart, Lung, and Blood Institute, [4]Genomic Core Laboratory, National Institute of Diabetes and Digestive and Kidney Diseases, [5]Laboratory of Human Carcinogenesis, Center for Cancer Research, National Cancer Institute, National Institutes of Health, Bethesda, MD 20892, USA, [6]Department of Pathology, University of Pittsburgh, Pittsburgh, PA 15213, USA

*Corresponding Author

Phone: 8822 4997

Fax: 8822 2314

Email: cxdeng@umac.mo

Running title: Negative reciprocal regulation between Sirt1 and Per2

Key words: Sirt1, Per2, Liver, Aging, and Circadian

Supplementary Methods

Wound healing assay. Six-month-old mice were anesthetized with Avertin. The dorsum shaved and cleaned with alcohol. Two equidistant 1-cm full-thickness incisional wounds were made through the skin and panniculus carnosus muscle. Wounds were photographed at days 5 and 7 post-wounding.

Peripheral blood chemistry analysis. Peripheral blood of mice was collected into EDTA-coated tubes. Blood samples were sent to NIH clinical service for counting the different types of blood cells present in the samples.

β-galactosidase activity assay. Tissues were embedded in OCT compound, and 10 μm cryosections were prepared and fixed with 1% formalin. Mouse embryonic fibroblasts (MEFs) were fixed with 0.5% glutaraldehyde for 5 min at room temperature. Slides were then incubated at 37ºC overnight with a solution containing $K_4[Fe(CN)_6]$ $3H_2O$ and $K_3[Fe(CN)_{..}$ Afterwards, the slides were counterstained with FastRed.

Immunofluorescent staining. Paraffin-embedded tissues were sectioned with a Microm HE340E to 5 μm. The sections were cooked in Reveal Decloaker (Biocare Medical) to reduce background staining, stained with antibody against γ-H2AX (Millipore).

Human hepatocyte isolation and culture. Human tissues were collected with informed consent following ethical and Institutional guidelines through the Liver Tissue Cell Distribution System (University of Pittsburgh, Pittsburgh, PA), which is funded by NIH Contract #

HSN276201200017C. Human hepatocytes were isolated from specimens derived from patients undergoing scheduled liver resection. Residual tissues not needed for diagnostic purposes after hepatic resection were transported to the laboratory from the operating room in cold Eagle's Minimum Essential Medium (Lonza, Walkersville, MD) within 90 min of removal. Briefly, the major hepatic vessels were cannulated and sutured on the cut surface of the parenchyma. A 3-step perfusion by peristaltic pump was conducted, followed by mechanical tissue disruption with sterile scissors and filtration through stainless-steel mesh and/or gauze to produce a cellular suspension. Hepatocytes were enriched and digestive enzymes were removed by 3 sequential low-speed centrifugations (85–90 x g). Only suspensions with more than 85% cell viability were used for experiments. Cell suspensions (1 ml; 1 x 10^6 viable hepatocytes/ml) were plated in collagen-treated 6-well plates with supplemented Hepatocyte Maintenance Medium (Lonza). After overnight culture at 37ºC and 5% CO_2, plated hepatocytes were used for experiments.

Western blot. Western blot analysis was performed with Licor (Lincoln, NE), utilizing antibodies against SIRT1 (Cell Signaling Technology, Cat. No. 2428), Per2 (Santa Cruz Biotechnology, Cat. No. sc-7727), Per2 (Santa Cruz Biotechnology, Cat. No. sc-25363) and Per2 (Abcam, Cat. No. ab179813), Clock (Santa Cruz Biotechnology, Cat. No. sc-6927), Bmal1 (Santa Cruz Biotechnology, Cat. No. sc-8550), Myc-tag (Santa Cruz Biotechnology, Cat. No. sc-40), p53 (Santa Cruz Biotechnology, Cat. No. sc-100), p16 (Santa Cruz Biotechnology, Cat. No. sc-1207), p21 (Santa Cruz Biotechnology, Cat. No. sc-471), β-actin (Sigma, Cat. No. A-5316), Nampt (Novus, Cat. No. NB100-594), histone H3 (Santa Cruz Biotechnology, Cat. No. sc-8654), histone H4 (Santa Cruz Biotechnology, Cat. No. sc-10810), histone H4K16ac (Santa Cruz Biotechnology, Cat. No. sc-8662-R), and histone H3K9ac (Millipore, Cat. No. 06-599). Staining

with actin was used as a loading control.

Primers.

ChIP primers

Name	Forward 5'-3'	Reverse 5'-3'
mPer2 +500	AGTGGTCCTTCCCCCAGGGC	CGCCGACTCCCATGGTGCTG
mPer2 -500	GGCCCCTCTGGATCTGCTGC	AATGACGGTCAGCCTGGGGG
mPer2 9194-9431	CTGGACCCATCCTAGGAACA	GACCCTTTTCCAGGTTCACA
mPer2 8792-9029	TTACAGAAACCCTGGCAACC	CATCCTCCACGCCAGTATTT
mUsp2 3900-4150	TCTGAATCCCCGAGATGTTC	GGGATTAAGAGGCCAAGAGC
mUsp2 4850-5050	CCTGGCCCTCTTAAGTTGTG	TCTTTCCCAAAGGGACAGTG
mSirt1 3-180	TCTTCCAACTGCCTCTCTGG	GTGACCCGGCGTGTTGTG

qRT-PCR primers

Name	Forward 5'-3'	Reverse 5'-3'
mPer2	AACAAATCCACCGGCTACTG	CTCCGGTGAGACTCCTCTTG
mKcnk5	ACCATCGGTTATGGCAATGT	TGGTAAGAAACTGGCCCAAC
mC2cd2l	GACAACCGTGCTGAGTGAGA	TCCTCTTGGTGGGTGTCTTC
mTle2	GCAGCTCTGTCGTGTATGGA	TGTGCCTACCAGAGCATCAG
mNapb	CGAGGAAATGTTTCCAGCAT	CCCTTGGATGGACTTCTTGA
mGermin	TGACCAATCTTGGCACACAT	AGGATTCGTCAGGCAGCTTA
mCrtc1	GCTGGAGCAGTTCAACATGA	CTGTCACCGTGAGGATGATG
mUsp2	GAACCAGCAAGCTCACAACA	CATTGAAAGTGTGCCATTCG
mTspan6	AAATGAGAAGGCCACCAATG	TCGCGTACAGTTTCAGCATC
mCyp7b1	CCTGCAGTCAACAGGTCAAA	TGCTGGAGTATGAGCACAGC
mPcmtd2	CTTCATCAGAACCAGCGACA	ATGACAAGGATGCCTCCAAC
mCreb3l2	AAGGTCTCTCGAACCTGCAA	TTGGTGGCAGAAGGATAAGG
mPer1	TGAAGCAAGACCGGGAGA	CACACACGCCATCACATCAA
mCry1	CTGAAGCAAAAATCGCCACCT	CACTGGTTCCGAAAGGGACTC
mCry2	TGGGCATCAACCGATGGA	CCCATTCCTTGCACAGCCTTG
mp16	CCGCTGCAGACAGACTGG	TGAGCAGAAGAGCTGCTACG
mp19	GCTCTGGCTTTCGTGAACAT	CGAATCTGCACCGTAGTTGA

qRT-PCR primers for human

	Forward 5'-3'	Reverse 5'-3'
hPER2-1	CTGGCCATCCACAAAAAGAT	CCTCCCAATGATGAAGGAGA
hSIRT1-1	GCAGATTAGTAGGCGGCTTG	TCTGGCATGTCCCACTATCA

Supplementary Figure Legend

Supplementary Figure 1 Deletion of *Sirt1* leads to premature aging. (A) Appearance of 12-month-old WT and MT mice. (B) Mean body weight (±SD) of WT and MT mice at different ages. (C) Mean number of granulocytes (±SD, 1000/µl) in peripheral blood analysis of WT and MT mice at different ages. For (B) and (C), all the experiments above, 3–5 mice per genotype were analyzed. * $p<0.05$. (D) Wound healing analyzed in WT and MT mice at 6 months of age. (E) β-galactosidase activity in tissue sections prepared from the kidney and brain of WT and MT mice at 6 months of age. (F) γH2AX foci formation in tissue sections prepared from the intestine and liver of WT and MT mice at 6 months of age. For panels D, E, and F, the results presented are representative of the animals analyzed in those experiments. (G) NAD+ level in wildtype (WT) and mutant (MT) mice at 3 months of age. (H) Quantification of western blot in figure 1F. (I) Quantification of western blot in figure 1G.

Supplementary Figure 2 Distribution of ChIP-Seq read densities of H4K16ac across the promoter regions of (A) Per2 and (B) Usp2. Highlighted are genomic regions that show an increase in H4K16ac read in MT mice when compared with WT mice. For each panel, the input and ChIP data are presented, the unit for the y-axis is RPBM (**R**ead **P**er **B**ase pair per **M**illion reads). Primer location is also indicated in panel A and B.

Supplementary Figure 3 Per2 and Sirt1 constitute a reciprocal negative regulation loop. (A) Western blot analysis of Per2 and Sirt1 protein expression in 3-month-old WT mouse liver obtained by circadian liver sample collection. Data are representative results from 3 mice. (B)

qRT-PCR analysis of *Sirt1* and *Per2* expression in 3-month-old WT mouse liver obtained by circadian liver sample collection. Data represent the mean (±SD, n=3) at each time point. The level of gene expression at ZT0 was set at 1. (C) Deletion analysis of *Sirt1* promoter activity using luciferase reporter activity assays in MEFs. Insert shows the E-box location within different lengths of the *Sirt1* promoter. (D) Effect of *Per2* expression on *Sirt1* promoter activity using luciferase reporter activity assays in MEFs. *$p<0.05$. Insert shows Per2 expression level. For C–D, 3 individual primary WT MEF cell lines were utilized; experiments were repeated twice in each line. Data represent the mean (±SD) for these experiments from one cell line. Luciferase activity level from GFP transfected samples was set as 1. (E) *SIRT1* promoter luciferase reporter activity of 202-Luc construct following ectopic expression of *Bmal1* (B), *Clock* (C), and *Per2* (P) in human HepG2 cells. Data represent the mean (±SD, n=3). (F) Biotin-labeled E-box sequences showing a possible Clock/Bmal1 binding site (100-116). (G) Biotin pull-down assays examining Bmal1/Clock/Per2 binding to the *Sirt1* promoter at E-box (100-116) region. The data are representative of experiments performed 3 times with independently prepared 293/HEK nuclear protein extracts. Mut, core domain mutated as shown in panel (F).

Supplementary Figure 4 Gene expression oscillation upon *Sirt1* deletion. (A) ChIP analysis of the binding of Bmal1, Clock, and Per2 to the *Sirt1* promoter upon serum shock and release in MEF cells. Inset legend indicates time interval after cells were returned to normal culture conditions 12 hours after serum shock. Three independent primary WT MEF cell lines were utilized. Data represent the mean (±SD) of 3 experiments using one cell line. * $p<0.05$. Each ChIP data was normalized with its own input, and then normalized again with IgG ChIP. Afterwards, ChIP value at 0h was set at 1. (B) qRT-PCR analysis of *Sirt1* expression in MEF

cells following serum shock. Three independent primary WT MEF cell lines were utilized. Data represent the mean (±SD) of 3 experiments using one cell line. Number of hours post-shock represents the time interval after cells were returned to normal culture conditions after serum shock. (C) qRT-PCR analysis of circadian-clock gene expression in MEF cells 16 h after cells were returned to normal culture conditions from serum shock. Three pairs of independent primary WT and MT MEF cell lines were utilized. Data represent the mean ± SD for one cell line. * $p<0.05$. Expression value at 12h in WT cells was set at 1. (D) qRT-PCR analysis of *SIRT1* and *PER2* expression in hTERT-immortalized human primary hepatocytes (HHT-4) subjected to shRNA knockdown of *SIRT1* (shT1) or *PER2* (shP) or overexpression of *SIRT1* (oxT1) or *PER2* (oxP). The level of expression for mock-transfected cells was set at 1. Experiments were repeated in HHT-4 cells for 3 times in triplicates. Data represent the mean (±SD) of one experiment. * $p<0.05$; ** $p<0.01$. Comparison was made between specific transfected sample vs GFP transfected sample. (E) ChIP of H4K16Ac on Per2 promoter 9194-9431 after SIRT1 overexpression in wildtype MEF cells. (F) ChIP of H4K16Ac on Per2 promoter 9194-9431 after SIRT1 knockdown in wildtype MEF cells. (G) qRT-PCR analysis of *Cry1* expression in the livers of 3-month-old WT or SIRT1LKO (MT) mice obtained by circadian liver sample collection. Data represent the mean (±SD, n=3). The level of gene expression for WT mice at ZT0 was set at 1. (H) qRT-PCR analysis of gene expression at ZT4 in the livers of 3-month-old WT or SIRT1LKO (LKO) mice obtained by circadian liver sample collection. Data represent the mean (±SD, n=3). *****$p<0.05$.

Supplementary Figure 5 Hepatic gene expression oscillation upon *Per2* overexpression or knockdown in mice. Mice at 3 months of age were used for the experiments. (A) qRT-PCR

analysis of hepatic *Per2* expression in WT mice after infected with virus for Flag-Per2, GFP, or shPer2. Data represent the mean (±SD, n=3). The level of gene expression for GFP lentivirus-infected mice was set as 1. * $p< 0.05$ represents the comparison between specific virus infected vs GFP virus infected. (B–C) qRT-PCR analysis of G6pase (B) and p19 (C) expression upon Per2 virus infection. The level of gene expression for GFP lentivirus-infected mice at ZT0 was set as 1. Data represent the mean (±SD, n=3). *p<0.05 when comparison was made between GFP cohort and Per2 overexpressed cohort. (D) Western blot and qRT-PCR validation of hepatic *Sirt1* and *Per2* expression in SIRT1LKO mice after infected with retrovirus for shLuc or shPer2. Data in lower panel represent the mean (±SD, n=3). The comparison was made against WT. The expression value from WT was set as 1. *p<0.05. (E–F) qRT-PCR analysis of G6pase (E) and p19 (F) expression from livers post lentivirus infection. WT, wild-type; LKO/shLuc, SIRT1LKO mice infected with shLuciferase lentivirus; LKO/shPer2, SIRT1LKO mice infected with shPer2 lentivirus. The level of gene expression of the WT mice at ZT0 was set as 1. Data represent the mean (±SD, n=3). *p<0.05 represents the comparison between LKO/shLuciferase and LKO/shPer2.

Supplementary Figure 6 Hepatic HIF-1α and its downstream signaling genes in WT and MT mice. (A) The total hepatic intracellular HIF-1α level was determined by Western blot. Upper panel compares protein expression in livers obtained during circadian liver sample collection from 3-month-old WT mice. MEF cell samples treated with or without DFX (DFX and C, respectively) were run on the same gel (with one well left empty between the MEF and liver samples) and served as a positive control. Middle panel compares liver HIF-1α expression in 3-month-old WT and MT mice. Lower panel compares liver HIF-1α expression in 3-month-old and 22-month-old WT mice. (B) Gene-expression levels of HIF-1α downstream genes in 3-

month-old WT, 3-month-old MT, 12-month-old WT, and 19-month-old WT mice, obtained by microarray analysis.

Supplementary Figure 1

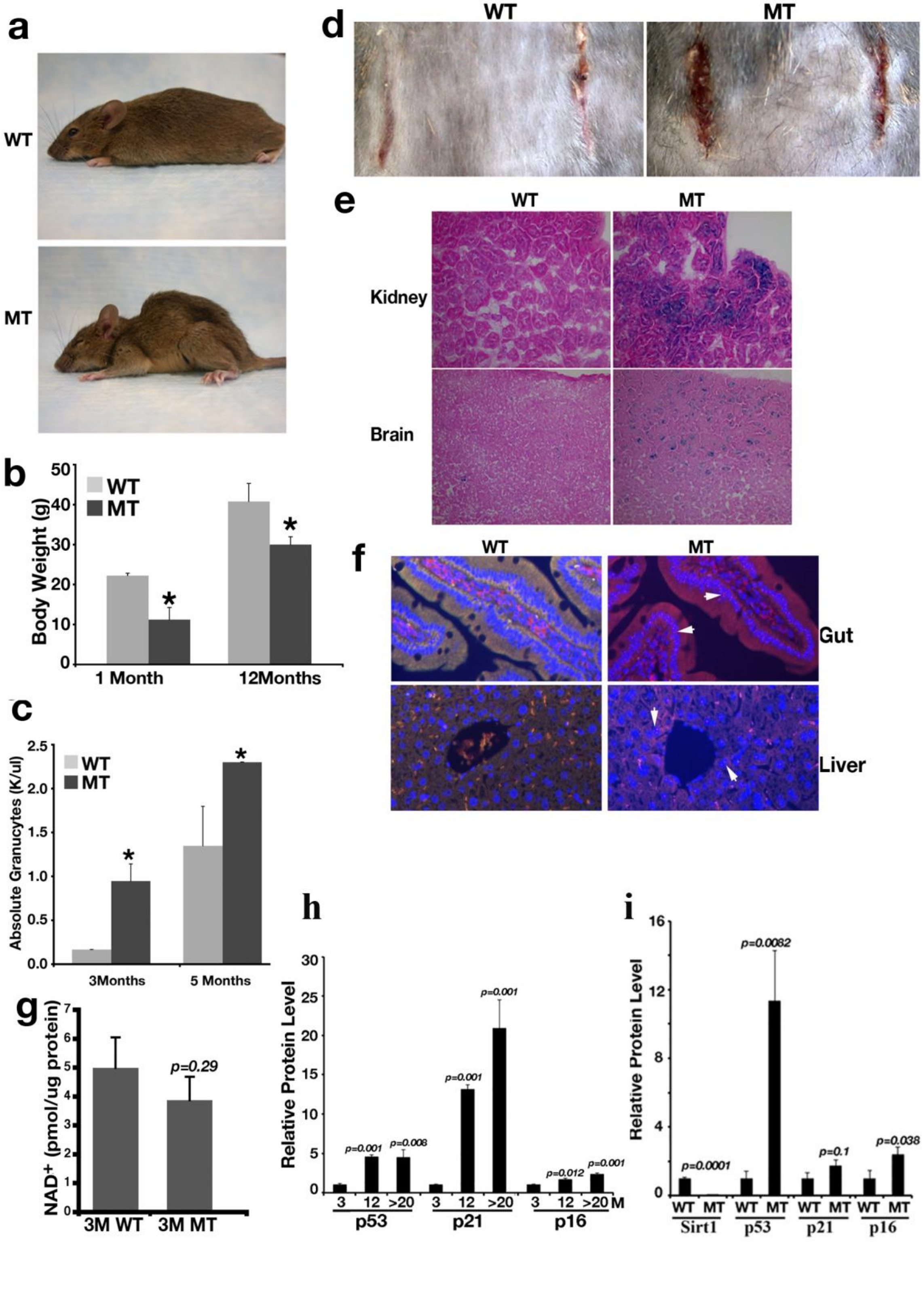

Supplementary Figure 2

a

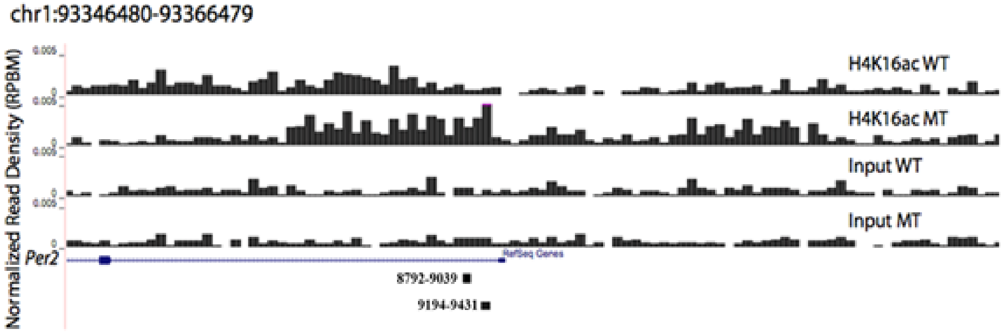

b

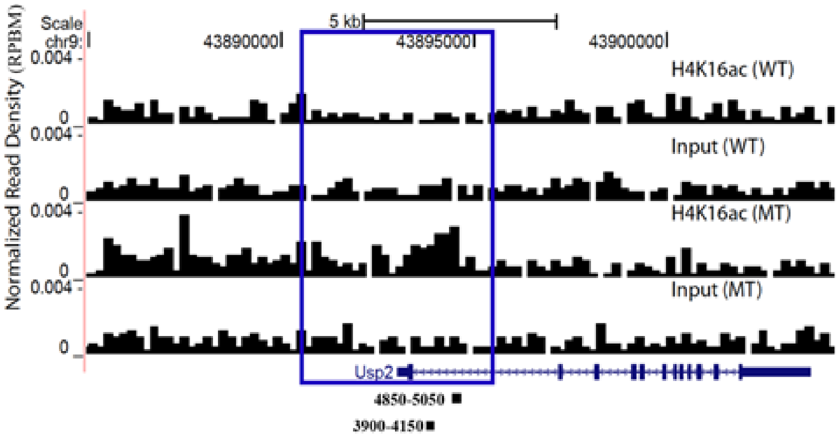

Supplementary Figure 3

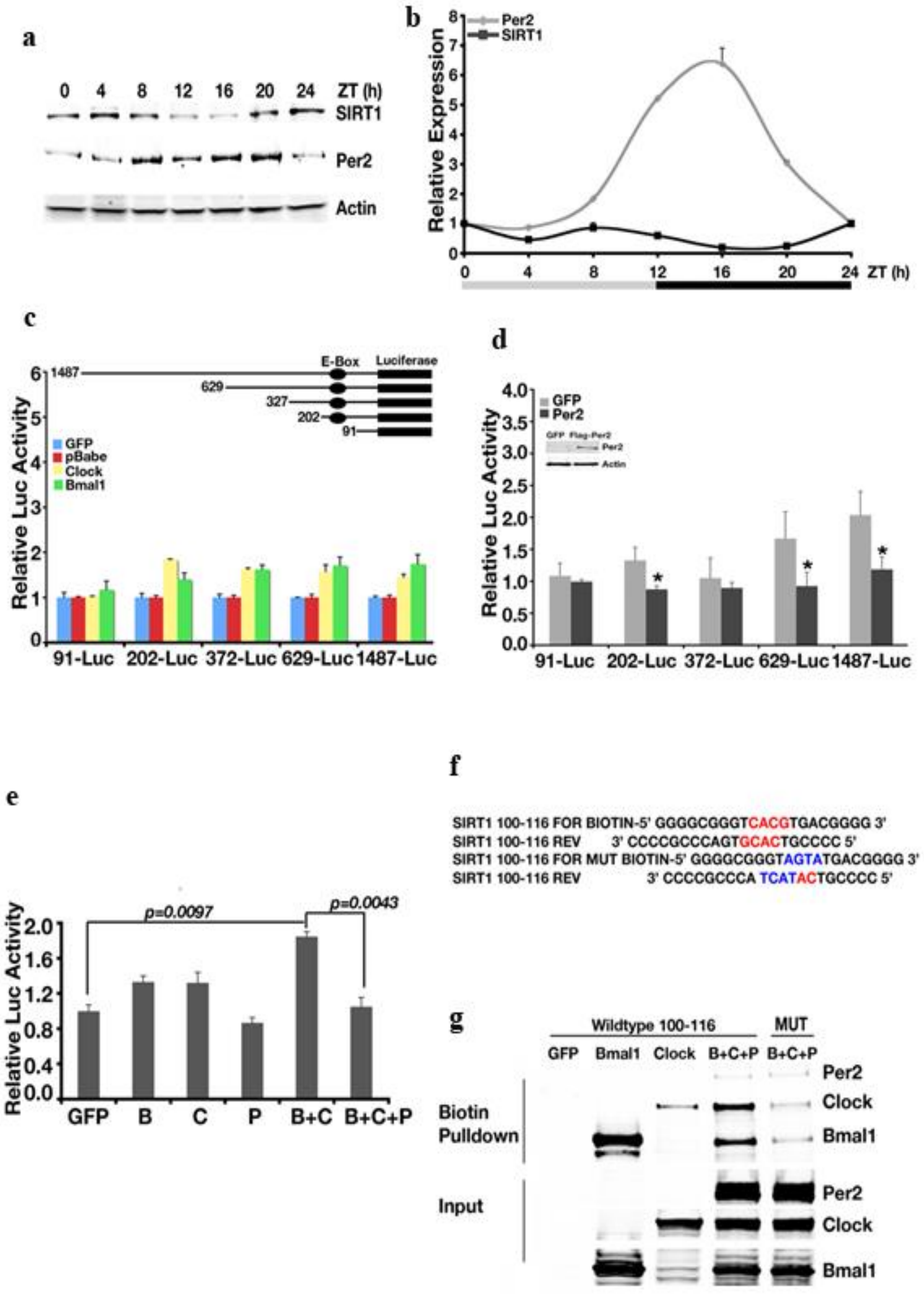

Supplementary Figure 4

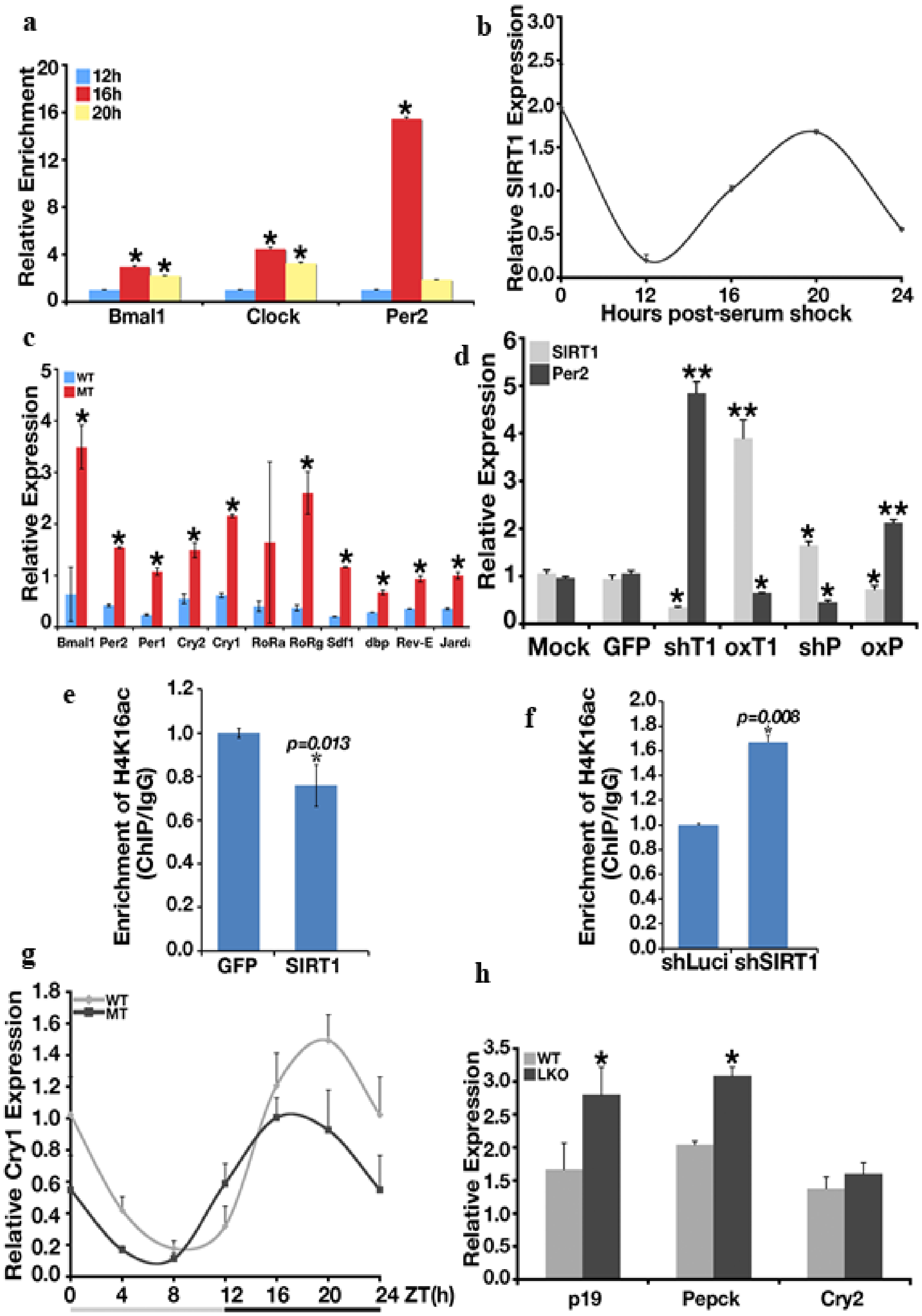

Supplementary Figure 5

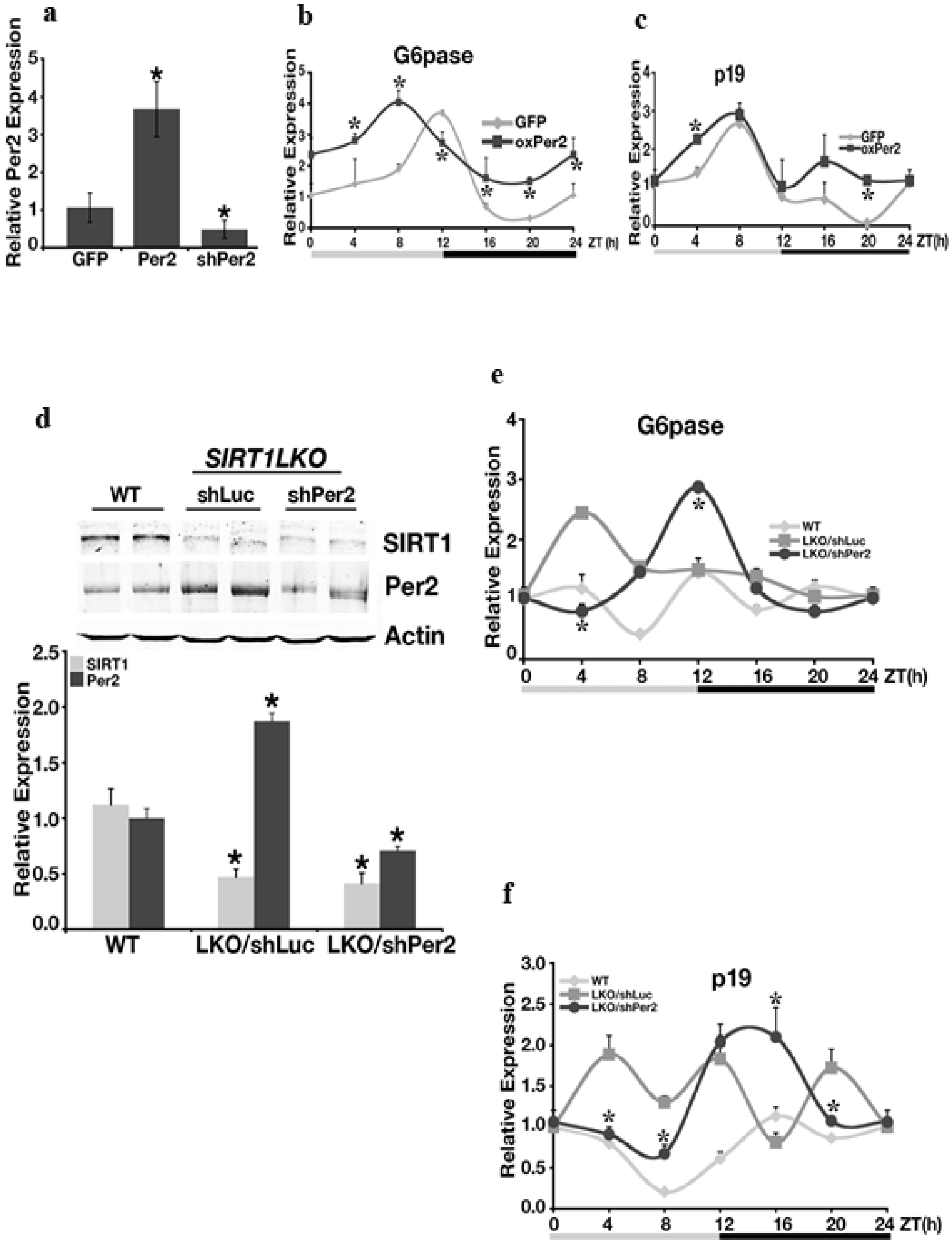

Supplementary Figure 6

a

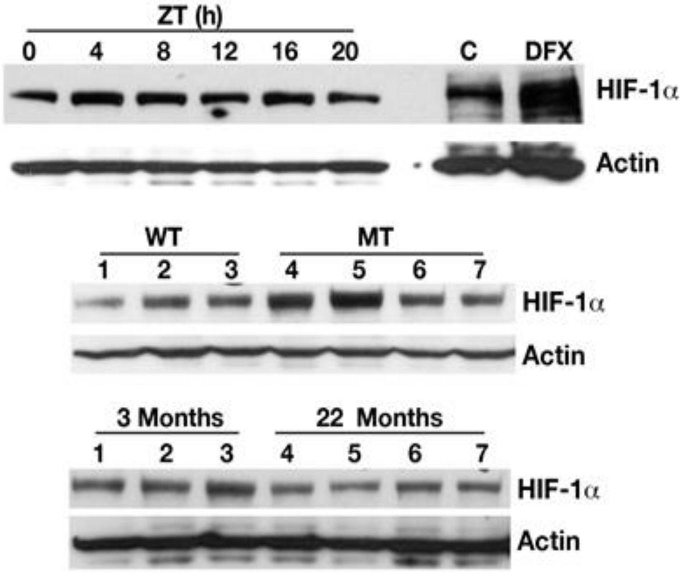

b

Microarray data for HIF1alpha downstream genes

Gene Symbol	3Mon WT	3Mon MT	12Mon WT	19Mon WT
Tfrc	6.07827365	6.66331555	5.793774	6.0429803
Fgf2	3.41478345	3.10976355	3.7409062	2.82965255
Igfbp3	10.434397	10.4841535	10.174875	10.04182
Igf2	5.197262	5.2802195	2.7804895	3.87094115
Kcnip3	4.06110335	4.027688	3.71244785	3.9268466
Tgfb3	5.15953835	5.340373	3.7439785	3.3212815
Cited2	10.8288645	9.77016975	10.3159685	10.471377
Ak3	9.672012	9.767706	9.7963305	9.7237175
Pgk1	13.4366285	13.32929825	13.359876	13.214354
Car9	6.81532475	4.48544695	6.5484328	6.0749755
Ldha	14.193981	14.29840425	14.1220035	14.2511475
Tfrc	9.1246885	9.5870655	9.310064	9.1717825
Flt1	7.29981325	7.50029515	6.4698331	6.6624054
Myt1	5.0233841	3.09713375	2.3053674	4.2357894
EG103324 /// EG4331	3.83860305	5.1518984	5.2798841	2.37890495
BC004004	9.3138895	8.8603935	9.493732	9.3332325
Hk2	6.8682645	6.99430535	7.1395262	7.30154535
Svs5	3.98514855	5.4605255	3.9001984	4.36775535
P4ha1	6.4211515	6.3704865	5.3740885	6.40960185
Nos2	2.12147295	2.18984185	1.97021675	2.10788355
Slc2a13	4.60239875	5.7345818	5.539965	6.112009
Slc2a2	14.3809415	14.52582	14.5439155	14.4382525
Slc2a3	6.2580375	5.94653155	5.41352885	5.88831995
Adra1b	7.616791	7.5114951	7.60592675	8.13463535
Adra1b	7.616791	7.5114951	7.60592675	8.13463535
Cadm1	9.9401565	10.1842175	9.494387	9.982355
p21	7.1402335	8.020135	7.52597975	7.437424
IGFBP1	13.887189	12.53709	12.736018	12.2427405
IGFBP2	14.30519525	13.8698845	12.261749	13.318667
VEGFb	7.74987135	7.87389275	8.0257837	8.5323455
PDK1	10.200607	9.952988	11.189682	9.467443
Gapdh	7.07766065	7.30191515	7.13793115	6.8228829

Supplementary Table 1
Up-regulated Aging Related Gene List in Log2 scale

Probe Set ID	FoldChange	Gene Symbol	RefSeq Transcript ID
1421653_a_at	3.33854875	Igh /// Igh-2	
1417602_at	1.991362	Per2	NM_011066
1430382_at	1.6320655	4833413G10Rik	
1451727_at	1.5806064	Slu7	NM_148673
1452073_at	1.4374072	6720460F02Rik	NM_144526
1417168_a_at	1.32626985	Usp2	NM_016808
1448945_at	1.28753525	Pllp	NM_026385
1427081_at	1.210118	A630072M18Rik	
1433383_at	1.11101025	1500002K03Rik	
1433622_at	1.02336125	Gemin4	NM_177367
1447724_x_at	0.9974115	Opa3	NM_207525
1452154_at	0.99717	Iars	NM_172015
1424081_at	0.9900115	Pcgf6	NM_027654
1421852_at	0.9851945	Kcnk5	NM_021542
1417904_at	0.926253	Dclre1a	NM_018831
1426532_at	0.9108435	Zmynd11	NM_144516
1427665_a_at	0.90785985	Nfic	NM_008688
1425019_at	0.90429525	Ubxn2a	NM_145441
1428543_at	0.89815805	Ppat	XM_001002879
1425060_s_at	0.8851545	Clip1	NM_019765
1425348_a_at	0.87796195	Srprb	NM_009275
1428788_at	0.8387275	Pgp	NM_025954
1416773_at	0.8262362	Wee1	NM_009516
1426607_at	0.82495475	EG633640	NM_001039244
1423795_at	0.816878	Sfpq	NM_023603
1417750_a_at	0.8008645	Slc25a37	NM_026331
1418133_at	0.7956235	Bcl3	NM_033601
1419751_x_at	0.771343	AB056442	XM_922697
1422767_at	0.76625675	Bysl	NM_016859
1452831_s_at	0.7409805	Ppat	XM_001002879
1448484_at	0.7218165	Amd1	NM_009665
1434149_at	0.7117385	Tcf4	NM_001083967
1422624_at	0.694453	Rev1	NM_019570
1428095_a_at	0.658932	C2cd2l	NM_027909

1434072_at	0.631109	Smcr7	NM_001009927
1439036_a_at	0.62932425	Atp1b1	NM_009721
1436244_a_at	0.6259381	Tle2	NM_019725
1448175_at	0.625829	Ehd1	NM_010119
1452940_x_at	0.61396015	Pitpnc1	NM_145823
1451167_at	0.613618	Ccdc101	NM_029339
1426835_at	0.612829	Metap1	NM_175224
1424019_at	0.6007355	Nop2	NM_138747
1424907_a_at	0.59755975	Farsa	NM_025648
1451152_a_at	0.592111	Atp1b1	NM_009721
1454680_at	0.58880025	D5Ertd579e	NM_001081232
1415860_at	0.588037	Kpna2	NM_010655
1415863_at	0.587991	Eif4g2	NM_001040131

Down-regulated Aging Related Gene List (Log2 scale)

**Probe Set ID**	**Fold Change**	**Gene Symbol**	**RefSeq Transcript ID**
1426065_a_at	-1.6149477	Trib3	NM_175093
1458284_at	-1.5547405	Ptbp1	NM_001077363
1423693_at	-1.44138825	cela1	NM_033612
1421075_s_at	-1.34913	Cyp7b1	NM_007825
1424029_at	-1.32843885	Tspyl4	NM_030203
1420836_at	-1.194633	Slc25a30	NM_026232
1421074_at	-1.192674	Cyp7b1	NM_007825
1428651_at	-1.162781	Klhl24	NM_029436
1440825_s_at	-1.0829895	Ccdc28a	NM_144820
1448839_at	-1.00542045	Kank3	NM_030697
1431234_at	-0.94888085	Itpripl1	XM_485065
1456174_x_at	-0.932915	Ndrg1	NM_008681
1438081_at	-0.9219625	Mcc	NM_001085373
1441124_at	-0.8633715	Vezt	NM_172538
1416872_at	-0.83546355	Tspan6	NM_019656
1422701_at	-0.8343735	Zap70	NM_009539
1428513_at	-0.826888	Calcoco1	NM_026192
1417811_at	-0.826539	Slc24a6	NM_133221
1434606_at	-0.804616	Erbb3	NM_010153
1453289_at	-0.78706485	Eif2c4	NM_153177
1449303_at	-0.781886	Sesn3	NM_030261
1452973_at	-0.7702215	Ppm1k	NM_175523
1443017_at	-0.756989	Cpeb2	NM_175937
1429418_at	-0.7334115	Cdc14b	NM_001122989
1420723_at	-0.7328895	Vnn3	NM_011979
1448318_at	-0.731294	Adfp	NM_007408
1449817_at	-0.721007	Abcb11	NM_021022

1437396_at	-0.7092305	Creb3l2	NM_178661
1460318_at	-0.690587	Csrp3	NM_013808
1415992_at	-0.6797015	Pigo	NM_020035
1452262_at	-0.677617	Grpel2	NM_021296
1416839_at	-0.6639235	Mut	NM_008650
1443822_s_at	-0.6624	Cisd1	NM_134007
1435355_at	-0.6480885	Neb	NM_010889
1419068_at	-0.62796975	Rabgef1	NM_019983
1436186_at	-0.61073515	E2f8	NM_001013368
1450408_at	-0.6092779	Clcn7	NM_011930
1421140_a_at	-0.5979305	Foxp1	NM_053202
1427075_s_at	-0.5951355	Pcmtd2	NM_153594
1451703_s_at	-0.59381125	Aprt	NM_009698

Supplementary Table 2

Up-regulated SIRT1 Dependent Aging Related Genes in Log2 scale

Probe Set ID	Fold Change	Gene Symbol	RefSeq Transcript ID
1417168_a_at	4.52525115	Usp2	NM_016808
1417602_at	3.6385435	Per2	NM_011066
1417904_at	1.9767065	Dclre1a	NM_018831
1421653_a_at	0.9542575	Igh	
1454680_at	0.948635	D5Ertd579e	NM_001081232
1436244_a_at	0.84533275	Tle2	NM_019725
1421852_at	0.811119	Kcnk5	NM_021542
1447724_x_at	0.7921775	Opa3	NM_207525
1419751_x_at	0.7685455	AB056442	XM_922697
1418133_at	0.765052	Bcl3	NM_033601
1433622_at	0.68437525	Gemin4	NM_177367
1428095_a_at	0.6575575	C2cd2l	NM_027909
1434072_at	0.5955415	Smcr7	NM_001009927
1459274_at	1.13001015	Gpr135	NM_181752
1450888_at	0.8469475	Napb	NM_019632
1438359_at	1.005751	Crtc1	NM_001004062

Down-regulated SIRT1 Dependent Aging Related Genes in Log2 scale

Probe Set ID	Fold Change	Gene Symbol	RefSeq Transcript ID
1421075_s_at	-2.0288475	Cyp7b1	NM_007825
1456174_x_at	-1.78116	Ndrg1	NM_008681
1443017_at	-1.1646335	Cpeb2	NM_175937
1453289_at	-1.0622048	Eif2c4	NM_153177
1437396_at	-0.99196595	Creb3l2	NM_178661

1438081_at	-0.926142	Mcc	NM_001085373
1416872_at	-0.8191034	Tspan6	NM_019656
1427075_s_at	-0.8154145	Pcmtd2	NM_153594

Supplementary Table 3

Validation of SIRT1 dependent aging related genes by qRT-PCR

Up-regulated genes

	**3mon WT**	**12monWT**	**19monWT**	**3mon MT**
per2	0.946±0.03	3.68±0.05	2.485±0.1	4.12±0.1
AB056442	1.025±0.09	2.79±0.14	3.37±0.2	3.487±0.18
Kcnk5	1±0.04	2.09±0.14	1.91±0.08	1.99±0.1
C2cd2l	1.012±0.08	1.91±0.12	2.58±0.07	1.633±0.03
Tle2	1.014±0.07	2.4±0.06	1.59±0.07	1.96±0.07
Napb	1.019±0.08	1.81±0.09	1.578±0.15	2.518±0.18
Gemin4	1.0039±0.04	1.7±0.03	4±0.15	2.37±0.04
Smcr7	1.026±0.1	1.55±0.1	4.27±0.17	3.12±0.07
2010003K15Rik	1.031±0.1	2.44±0.02	4.797±0.13	2.229±0.09
Ctrc1	1.019±0.08	2.047±0.17	2.594±0.8	1.86±0.04
Opa3	1.294±0.29	3.5±0.35	2.03±0.16	1.62±0.14
D5Ertd579e	1.023±0.08	3.973±0.07	2.384±0.02	3.442±0.13
Usp2	1.019±0.08	4.676±0.57	3.72±0.96	30.76±5.86
Gpr135	1.016±0.077	2.709±0.25	2.466±0.17	1.146±0.05

Down regulated genes

	**3mon WT**	**12mon WT**	**19mon WT**	**3mon MT**
Tspan6	1.001±0.02	0.833±0.08	0.615±0.01	0.681±0.03
Cyp7b1	1.06±0.14	0.686±0.07	0.573±0.13	0.139±0.06
Pcmtd2	1.01±0.06	0.634±0.03	0.454±0.08	0.525±0.01
Creb3l2	1.016±0.08	0.825±0.02	0.188±0.05	0.619±0.06
Ndrg1	1.063±0.16	0.162±0.04	0.098±0.04	0.157±0.03
MCC	1.031±0.1	0.678±0.04	0.522±0.07	0.435±0.02
Cpeb2	1.062±0.105	0.533±0.179	0.253±0.09	0.598±0.05
Eif2c4	1.008±0.05	0.841±0.01	0.536±0.01	0.588±0.03
Clock	1.018±0.08	1.085±0.24	0.842±0.23	0.431±0.02
